# Characterization of Genomic, Physiological, and Probiotic Features of *Lactiplantibacillus plantarum* JS21 Strain Isolated from Traditional Fermented Jiangshui

**DOI:** 10.3390/foods13071082

**Published:** 2024-04-01

**Authors:** Yang Liu, Shanshan Wang, Ling Wang, Hongzhao Lu, Tao Zhang, Wenxian Zeng

**Affiliations:** 1 School of Biological Science and Engineering, Shaanxi University of Technology, Hanzhong 723001, China; ly_022599@163.com (Y.L.); zengwenxian@snut.edu.cn (W.Z.); 2 QinLing-Bashan Mountains Bioresources Comprehensive Development C. I. C., Shaanxi University of Technology, Hanzhong 723001, China; 3 Engineering Research Center of Quality Improvement and Safety Control of Qinba Special Meat Products, Shaanxi University of Technology, Hanzhong 723001, China; 4 Shaanxi Union Research Center of University and Enterprise for Zhenba Bacon, Shaanxi University of Technology, Hanzhong 723001, China; 5 Qinba State Key Laboratory of Biological Resources and Ecological Environment, Shaanxi University of Technology, Hanzhong 723001, China; 6 Shaanxi Province Key Laboratory of Bio-Resources, Shaanxi University of Technology, Hanzhong 723001, China

**Keywords:** Jiangshui, *Lactiplantibacillus plantarum*, genome, probiotic, bacteriocin

## Abstract

This study aimed to understand the genetic and metabolic traits of a *Lactiplantibacillus plantarum* JS21 strain and its probiotic abilities through laboratory tests and computer analysis. *L. plantarum* JS21 was isolated from a traditional fermented food known as “Jiangshui” in Hanzhong city. In this research, the complete genetic makeup of JS21 was determined using Illumina and PacBio technologies. The JS21 genome consisted of a 3.423 Mb circular chromosome and five plasmids. It was found to contain 3023 protein-coding genes, 16 tRNA genes, 64 rRNA operons, 40 non-coding RNA genes, 264 pseudogenes, and six CRISPR array regions. The GC content of the genome was 44.53%. Additionally, the genome harbored three complete prophages. The evolutionary relationship and the genome collinearity of JS21 were compared with other *L. plantarum* strains. The resistance genes identified in JS21 were inherent. Enzyme genes involved in the Embden–Meyerhof–Parnas (EMP) and phosphoketolase (PK) pathways were detected, indicating potential for facultative heterofermentative pathways. JS21 possessed bacteriocins *plnE*/*plnF* genes and genes for polyketide and terpenoid assembly, possibly contributing to its antibacterial properties against *Escherichia coli* (ATCC 25922), *Escherichia coli* (K88), *Staphylococcus aureus* (CMCC 26003), and *Listeria monocytogenes* (CICC 21635). Furthermore, JS21 carried genes for Na^+^/H^+^ antiporters, F_0_F_1_ ATPase, and other stress resistance genes, which may account for its ability to withstand simulated conditions of the human gastrointestinal tract *in vitro*. The high hydrophobicity of its cell surface suggested the potential for intestinal colonization. Overall, *L. plantarum* JS21 exhibited probiotic traits as evidenced by laboratory experiments and computational analysis, suggesting its suitability as a dietary supplement.

## 1. Introduction

Lactic acid bacteria are a type of bacteria that ferment carbohydrates to produce lactic acid. They are found in various sources, including humans, animals, plants, and the environment [1]. These microorganisms are essential for maintaining a balanced microbial environment within the body, preventing harmful bacteria from colonizing, and protecting the integrity of mucosal barriers [2]. Additionally, lactic acid bacteria enhance mucosal immunity and the body’s defenses against bacterial infections [3,4]. The antimicrobial peptides produced by these bacteria, known as bacteriocins, have low potential for inducing drug resistance, making them promising candidates for clinical anti-infection treatments [5,6]. Due to their potential probiotic properties, many lactic acid bacteria have been acknowledged as generally recognized as safe (GRAS) and/or presumably qualified as safe (QPS). Probiotics, as defined by the International Scientific Association for Probiotics and Prebiotics (ISAPP), are harmless living microorganisms that provide health benefits to the host when consumed in sufficient quantities [7].

Jiangshui is a traditional fermented vegetable beverage in Northwest China with a history spanning over two millennia, created through microbial fermentation without heat treatment. It has a pale and clear appearance with a mildly acidic yet rich taste, serving as a refreshing beverage or a culinary enhancer in various dishes like stir-fries and soups. Jiangshui is rich in probiotics, vitamin C, organic acids, and nutrients, which contribute to its digestive promotion, cholesterol reduction, and support for intestinal health [8,9,10]. Lactic acid bacteria play a crucial role in Jiangshui fermentation, making it a viable source for isolating bacteria [11,12]. Bacterial strains derived from plants are generally considered to have a safer profile compared to those from animal sources [13,14]. However, further investigation is needed to understand the adaptation of plant-derived bacteria to the human gut and their probiotic effectiveness [15]. Probiotic screening involves assessing tolerance to gastric acidity, bile salts, antagonistic activity against pathogens, and antibiotic susceptibility [16]. Ou et al. [17] reported the isolation of *L. plantarum* from Jiangshui, confirming its probiotic safety through extensive *in vitro* and *in vivo* toxicological tests. Wu et al. [18] found that *Limmosilactobacillus fermentum* JL-3, isolated from Jiangshui, significantly reduced uric acid levels in mouse models. However, research on probiotics in Jiangshui has mainly focused on microbial diversity and phenotypic functions, with few studies exploring functional genes based on strain genomes.

*L. plantarum* has been widely utilized in various fields such as fruit and vegetable preservation, feed production, dairy, and meat fermentation, due to its ability to suppress spoilage bacteria, enhance food palatability, and prevent chronic metabolic disorders, cholesterol elevation, immune function impairment, and oxidative stress [19,20,21]. Shi et al. [22] found that *L. plantarum* SC-5 alleviated clinical symptoms and restored intestinal flora balance in colitis mice. Yu et al. [23] found that oral administration of the probiotic *L. plantarum* FLPL05 maintained the integrity of the intestinal mucosal barrier in naturally aged mice, reduced the level of inflammation, and prolonged their lifespan. In our previous study, we isolated the *L. plantarum* JS21 strain from Jiangshui. However, the genomic information and physiological functions of JS21 remained unclear.

This study aimed to assess the genomic characteristics and probiotic properties of *L. plantarum* JS21 using *in vitro* and *in silico* methods. The whole genome of JS21 was analyzed using bioinformatics tools. Additionally, the physiological properties of the strain were examined based on probiotic screening and selection criteria. Finally, we explored the probiotic functional genes related to the physiological properties of JS21. These findings elucidated the genomic and metabolic traits of *L. plantarum* strain JS21 isolated from Jiangshui, laying the groundwork for its potential application as a probiotic.

## 2. Materials and Methods

### 2.1. Isolation of Bacterial Strain and Growth Conditions

*L. plantarum* JS21 was isolated from Jiangshui in Hanzhong, China. The sample was gradientally diluted with sterile saline solution (0.85%). Then, 100 μL of each dilution was applied to MRS agar (Qingdao Hi-Tech Industrial Park Haibo Biotechnology Co., Ltd., Qingdao, China) and then incubated at 37 °C for 24–48 h. JS21 strain was isolated and purified from a 10^−5^ diluted sample. Purified JS21 was subjected to catalase detection and Gram staining. JS21 was preserved with 25% glycerol at −80 °C. The growth curve and pH curve were determined as follows. The activated strain was inoculated with 1% inoculum into MRS liquid medium and incubated at 37 °C. The OD_600_ was determined at 0, 1, 2, 3, 4, 5, 6, 7, 8, 9, 10, 11, 12, 16, 20, 24, and 28 h. The growth curve was plotted with the incubation time as the horizontal coordinate and OD_600_ as the vertical coordinate. The pH of the medium was measured at 0, 4, 8, 12, 16, 20, 24, 28, 40, and 52 h. The pH curve was plotted with the incubation time as the horizontal coordinate and pH as the vertical coordinate.

### 2.2. DNA Extraction, Identification, Whole Genome Sequencing, and Assembly

First, *L. plantarum* JS21 culture was subcultured twice in MRS broth followed by incubation anaerobically at 37 °C for 24 h. A 50-mL fresh culture was pipetted onto a sterile tube and centrifuged at 6000× *g* for 10 min at 4 °C. Genomic DNA was extracted using the Wizard^®^ Genomic DNA Purification Kit (Promega, Madison, WI, USA) according to the manufacturer’s protocol. Purified genomic DNA was quantified by TBS-380 fluorometer (Turner BioSystems Inc., Sunnyvale, CA, USA). High quality DNA (OD_260/280_ = 1.8~2.0, >20 μg) was used to carry out further research. Shanghai Majorbio Bio-pharm Technology Co., Ltd. (Shanghai, China) sequenced the whole genome of *L. plantarum* JS21 using two different sequencing techniques. The genome was sequenced using a combination of PacBio RS II Single Molecule Real Time (SMRT) and Illumina sequencing platforms. The Illumina data were used to evaluate the complexity of the genome.

### 2.3. Bioinformatic Analysis

Glimmer [24] was used for CDS prediction, tRNA-scan-SE (v2.0) was used for tRNA prediction, and Barrnap was used for rRNA prediction. The predicted CDSs were annotated from NR and the KEGG database using sequence alignment tools such as BLAST, Diamond, and HMMER. Genomic circle mapping was performed using Circos software (v0.69.6). A BLAST Ring Image of the genome of the JS21 strain and other compared *L. plantarum* strains was generated using BRIG v0.95 [25]. Prediction of CRISPR-Cas regions was implemented via the CRISPR finder online tool (http://crispr.i2bc.paris-saclay.fr/Server/, accessed on 10 August 2023). The eleven genome sequences of *L. plantarum* (Y44, WCFS1, SRCM100442, Q180, P9, MA2, LP3, LP-F1, DSM 20174), *L. pentosus* (DSM 20314), and *L. paraplantarum* (FL-8) isolated from different sources were acquired from NCBI for comparative genomic analysis. The average nucleotide identity values (ANIs) were analyzed using the online tool of the Majorbio Cloud Platform (https://cloud.majorbio.com/page/tools/, accessed on 31 August 2023). Collinearity of JS21, WCFS1, and DSM 20174 genomes was analyzed using the Mauve (Version: 1.1.3) plugin in Geneious Prime (version 2023.2.1) software. The prophage elements on the genome of JS21 were identified with the Phage Search Tool Enhanced Release (PHASTER) [26]. All protein-coding sequences obtained from PHASTER have been screened against the non-redundant protein (NR) database by performing protein BLAST to identify the horizontally transferred genes. If a gene’s homologous protein was found to match a microorganism other than *L. plantarum* by ≥80%, that gene was noted as horizontally transferred [27]. A resistome screening was conducted by scanning the complete genome sequence of the JS21 strain versus the ResFinder 4.3.1, CARD, and KEGG databases, respectively. The analysis was conducted with a specificity threshold of 90% identity and a minimum sequence length of 60% [28,29,30]. As with the phage elements, horizontal gene transfer screening was performed within the detected resistance genes. The secondary metabolite biosynthetic gene clusters of JS21 were detected using the Antibiotics and Secondary Metabolite Analysis Shell antiSMASH (version 7.0.1) program [31]. The whole genome sequence data reported in this paper have been deposited in the Genome Warehouse in National Genomics Data Center, Beijing Institute of Genomics, Chinese Academy of Sciences/China National Center for Bioinformation, under accession number GWHEROS00000000 that is publicly accessible at https://ngdc.cncb.ac.cn/gwh, accessed on 31 August 2023.

### 2.4. Carbohydrate Fermentation

The carbohydrate metabolism characteristic of JS21 was analyzed using the API 50 CHL kit (BioMérieux, Marcy l’Etoile, France).

### 2.5. Determination of Antibiotic Susceptibility

Drug susceptibility testing was performed using the Kirby–Bauer method to determine the resistance or susceptibility of the JS21 strain [32]. Commercial antibiotic disks (penicillin G, ampicillin, amoxicillin, cefotaxime, kanamycin, gentamicin, erythromycin, tetracycline, minocycline, ciprofloxacin, norfloxacin, enrofloxacin, vancomycin, and sulfafurazole (Hangzhou Microbial Reagent Co., Ltd., Hangzhou, China)) were used for antibiotic susceptibility testing of *L. plantarum* JS21. The interpretation of the inhibition zone (mm) was carried out as described by the judgment standard for the drug resistance of *Enterococcus* for NCCLS and the judgment standard for the drug resistance of *L. plantarum* for Charteris [33,34].

### 2.6. Determination of Probiotic Properties

In order to determine the probiotic properties of JS21, β-hemolysis, *in vitro* simulated gastrointestinal digestion, cell surface hydrophobicity, cell auto-aggregation experiments, and co-aggregation and antibacterial activity experiments against several pathogenic bacteria were carried out, respectively. The β-hemolytic activity was determined with a Columbia CNA Blood Agar Plate [35]. In order to test the resistance of JS21 to gastrointestinal digestion, simulated gastric [36] and intestinal fluids [37] were prepared according to the Chinese Pharmacopoeia Commission, respectively. Simulated gastric fluids with pH 2.0 and pH 3.0 were prepared, respectively. The pH of the simulated intestinal fluid was 6.8. The JS21 strain was inoculated into the simulated gastric fluids at 10% inoculum and incubated at 37 °C. The flat colony counting method was carried out at 0 h and 3 h. The inoculation after 3 h in the simulated gastric fluids was inoculated at 10% of the simulated intestinal fluid at 37 °C. The flat colony counting method was carried out at 0 h, 3 h, and 6 h, and then the survival rate was calculated. Cell surface hydrophobicity experiments were carried out with reference to the method of Liu et al. [38]. Auto-aggregation and co-aggregation experiments were performed according to the method of Behrooz et al. [39]. Well diffusion was performed to evaluate the inhibitory effects of lactic acid bacteria strains on the growth of indicator strains. *Escherichia coli* (ATCC 25922), *Escherichia coli* (K88), *Staphylococcus aureus* (CMCC 26003), and *Listeria monocytogenes* (CICC 21635) were used as indicator bacteria. The indicator strains were cultured and diluted to OD_600_ 0.5. Then, 50 μL of diluted strains was added into 50 mL of 0.8% LB agar, mixed thoroughly, and poured into the plate. Eight-millimeter wells were punched on plates using a sterile borer. Then, each well was filled with 300 μL of filtered supernatant and incubated overnight at 37 °C. The sizes of the inhibition zone were measured in millimeters. Meanwhile, in order to determine the ability of the cell-free supernatant (CFS) to inhibi the growth of pathogenic bacteria, the indicator bacteria were diluted to OD_600_ 0.5. The indicator bacteria, LB medium, and CFS were mixed in the ratio of 1:17:2, and were incubated in 96-well plates with shaking at 37 °C. The OD_600_ was measured per hour using a multi-function measuring instrument (TECAN Trading, Ltd., Shanghai, China), and the growth curve was plotted for 36 h.

## 3. Results and Discussion

### 3.1. Functional Genomic Characterization

**Figure 1:**
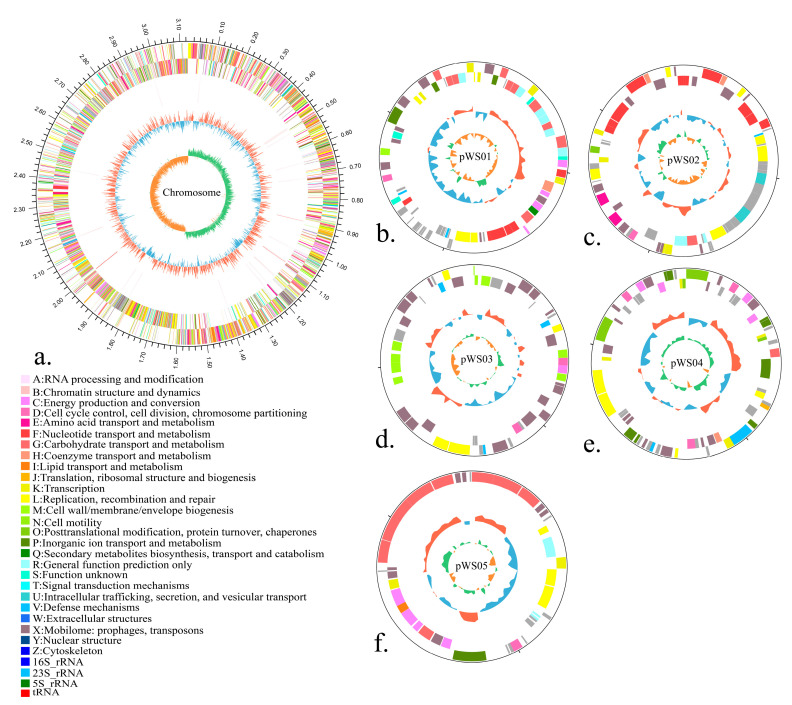
Graphical circular map of the chromosome (**a**) and plasmids (**b**–**f**) of *Lactiplantibacillus plantarum* JS21. Circles from outside to the center: genes on forward strand (color by COG categories); genes on reverse strand (color by COG categories); GC-content; GC skew.

**Figure 2:**
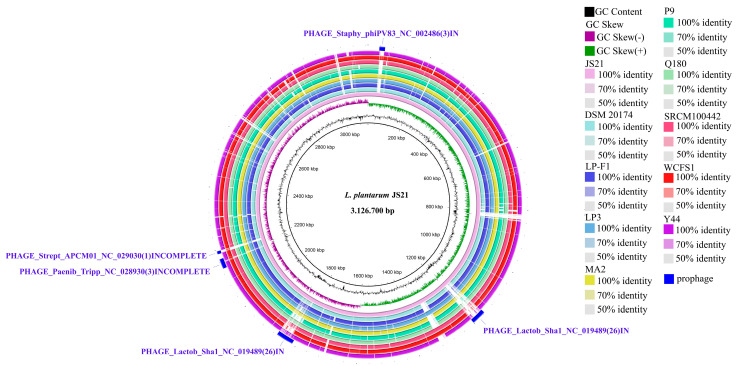
The BLAST Ring alignment of *Lactiplantibacillus plantarum* JS21 versus other compared well-known *Lactiplantibacillus plantarum* genomes, which are aligned from the outer side to the inside as Y44, WCFS1, SRCM100442, Q180, P9, MA2, LP3, LP-F1, DSM 20174, and JS21, respectively. The GC content is illustrated in the 2nd inner circle before the genome of JS21. The GC skew (+/−) of the genome of JS21 is also shown in the third inner circle, and in the first inner circle, the genome size is demonstrated. On the other hand, prophage regions of the strain JS21 are depicted as blue colored arcs in the outer circle. The numbers in parentheses showed the number of matched proteins of the phages. At the end of the phage names, IN indicates intact, and INCOMPLETE indicates incomplete.

The raw sequencing data were assembled into six contigs, resulting in a genome size of 3.423 Mb. The JS21 genome comprised a circular chromosome and five plasmids, with an overall GC content of 44.53%. Genome annotation identified 3023 protein-coding sequences (CDS), 16 transfer RNA (tRNA) genes, 64 ribosomal RNA (rRNA) operons, 40 non-coding RNA molecules, 264 pseudogenes, and six CRISPR array regions. Notably, the CRISPR Finder tool revealed the absence of Cas proteins within the genome (Figure 1, Table 1). Comparative genomic analysis highlighted genomic differences between JS21 and other *L. plantarum* genomes, identifying gaps (refer to Figure 2). These intergenic regions often represent genomic islands characterized by reduced GC content, suggesting potential horizontal gene transfer, typically containing integrases and transposases [40,41].

**Figure 3:**
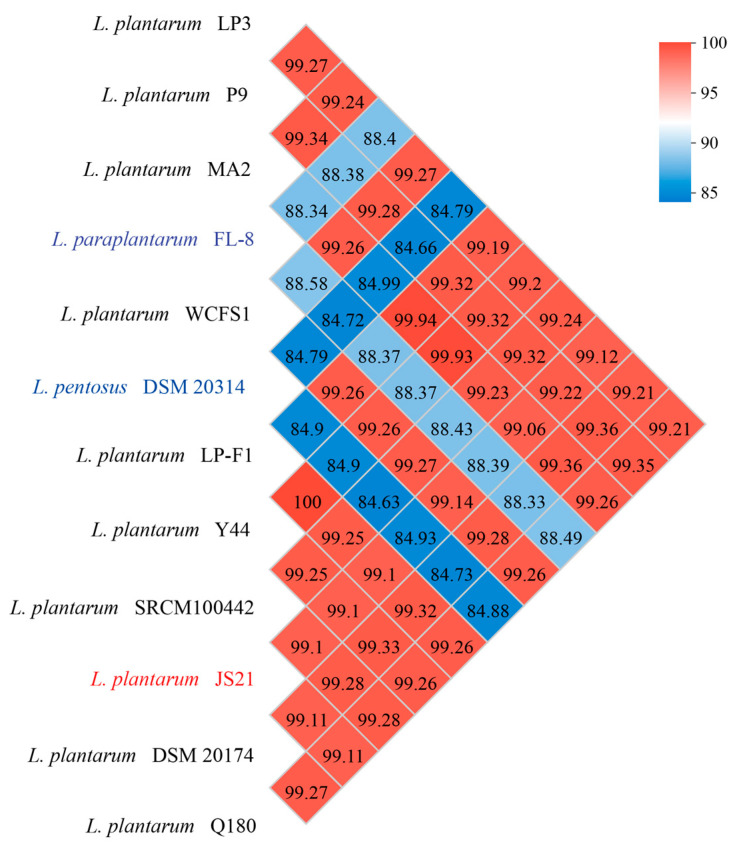
Average nucleotide identity (ANI) values of *Lactiplantibacillus plantarum* JS21 and other compared well-known *Lactiplantibacillus* species.

In order to compare the differences between strains from different sources, the ANI values for JS21 compared to other *L. plantarum* strains are illustrated in Figure 3. According to the ANI results, JS21 exhibited an identical genetic reciprocal similarity of 99.22% and 99.14% with strains P9 and WCFS1, respectively. P9 was isolated from traditional sour porridge in Inner Mongolia, while WCFS1 originated from human saliva and has been commercially utilized [42,43,44]. Furthermore, JS21 showed an identical genetic reciprocal similarity of 99.12% and 99.11% with strains LP3 and DSM 20174, respectively. LP3 was isolated from commercial dietary supplements by the Korean Academy of Food Sciences, and DSM 20174 was isolated from sauerkraut by the Helmhertz Institute in Germany [45]. Strains isolated from diverse ecological niches such as fermented milk (LP-F1), turbot fish (Y44), and infant feces (SRCM100442) exhibited slightly lower genetic similarities with JS21 [46,47]. Conversely, strains Q180 (99.08%) and MA2 (99.06%) displayed the lowest genetic similarity to JS21. This is expected, as strain Q180 was isolated from Korean adult feces, which exhibit greater genetic distance compared to other strains, and MA2 was derived from Kefir, a traditional Tibetan food, potentially explaining its lower genetic similarity due to its unique geographical origin [48,49,50]. The studies show that *L. plantarum* inhabits various environments with similar genetic characteristics to *L. pentosus* and *L. paraplantarum* [27]. The ANI analysis, commonly used to determine species membership, confirmed that strain JS21 belongs to *L. plantarum*, as the calculated ANI value comparing genomes of *L. plantarum*, *L. pentosus*, and *L. paraplantarum* was approximately 88%, significantly lower than the 95% cutoff score for species membership [51,52].

**Figure 4:**
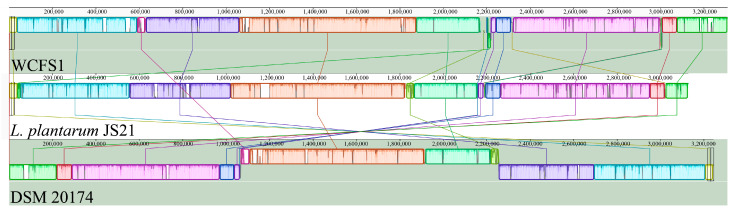
Multiple sequence alignment of the *Lactiplantibacillus plantarum* genomic.

Collinearity analysis was employed to investigate genome correlation by identifying homologous sequences and examining their arrangement consistency among different individuals of the same species [53]. WCFS1 and DSM 20174, because of their high ANI values, were selected for collinearity analysis with JS21 (Figure 4). The analysis revealed 14.402 kb locally collinear blocks (LCB) at 38.713 kb of the JS21 genome, exhibiting 78.8% identity with WCFS1 and a genomic translocation, which was absent in DSM 20174. Additionally, at 1.830 Mb of LCB, a 36.374 kb fragment showed good collinearity with DSM 20174, while WCFS1 exhibited numerous fragment deletions. A 3.075 kb fragment at 2.190 Mb was consistent with WCFS1, but a deletion was observed in DSM 20174. JS21 harbored unique fragments, including a 6.113 kb segment at genome position 1.824 Mb, containing genes encoding LysM peptidoglycan-binding domain-containing protein and *sadA* annotated as a phage tail protein. Furthermore, JS21 contained unique 2.394 kb fragments encoding the parA gene at genome position 2.156 Mb, possibly involved in DNA cleavage according to COG database annotation. These above sequence regions were located in predicted prophage regions based on annotation information. JS21 also possessed a 2.951 kb unique fragment at 3.019 Mb, containing the gene encoding α-galactosidase (*galA*). Moreover, a 1.353 kb unique fragment encoded the LacI family transcriptional regulator gene at 3.023 Mb. The results indicated good collinearity between JS21 and WCFS1, with significant genetic differences observed between JS21 and DSM 20174. Studies have suggested that, when a gene is located at the edge of a translocation region, it may be interrupted, leading to substantial changes in the DNA sequence [54].

### 3.2. Prophages, Related Horizontal Gene Transfer, and Mobilome

The search for prophages revealed the presence of five prophage regions in the JS21 genome, including three intact and two incomplete prophages, as summarized in Table S1. Details of each identified phage region are listed in Tables S2–S6. The intact prophage regions (region 1, region 2, and region 3) showed similarities to Staphy_phiPV83_NC_002486 (18.1 Kb), Lactob_Sha1_NC_019489 (46 Kb), and Lactob_Sha1_NC_019489 (55.4 Kb), respectively. Among the identified phages, Lactob_Sha1 displayed the highest protein match, making it the most common prophage in *L. plantarum* [55]. All phage regions contained *attL*/*attR* sequences and integrase genes. Integrase genes serve as functional markers for phages, pathogenicity islands, and integrative plasmids within bacterial genomes [27,40,56]. Three integrases [PP_00032 (region 1), PP_01076 (region 2), and PP_01828 (region 3)] were detected in the intact regions, while two integrases [PP_02114 (region 4) and PP_02152 (region 5)] were identified in the incomplete regions. It is noteworthy that the *attL* sequences were located upstream of the integrase genes in regions 1 and 2, whereas the integrase gene was situated downstream of the *attR* sequence in region 3. Both incomplete regions had integrase genes located upstream of the *attR* sequences. Additionally, genomic islands can be distinguished from the rest of the bacterial genome based on nucleotide statistics such as cumulative GC skew, GC% content, codon usage, or tetranucleotide frequencies [57]. Furthermore, *attL* and *attR* sequences were identical within each intact phage. No virulence or AMR genes were found within the intact prophages. Endolysin genes were annotated in phages 2 and 3. Endolysins are phage proteins that rapidly break down bacterial cell walls and release new phages [58]. This suggests that the identified phages could pose a potential threat to JS21.

Horizontal gene transfer (HGT) among bacteria commonly occurs through bacteriophage infection or natural competence [27,56]. Sequence homology screening results indicated that most of the phage genes in JS21 were similar to those in *L. plantarum*, with only 2.08% (71 phage genes) of the total genes in the genome likely acquired through HGT from other bacteria (Table S7). These sources include *Lactiplantibacillus argentoratensis*, *Lactiplantibacillus nangangensis*, *Lentilactobacillus buchneri*, *Levilactobacillus brevis*, *Lactiplantibacillus xiangfangensis*, *Limosilactobacillus reuteri*, *Caudoviricetes* sp., *Levilactobacillus fuyuanensis*, *Lactobacillus japonicus*, *Lactiplantibacillus mudanjiangensis*, *Weissella confuse*, *Weissella cibaria*, *Lactobacillus phage Sha1*, *Siphoviridae* sp. *ctk5O4*, *Bacteroides fragilis*, *Loigolactobacillus backii*, *Gluconobacter oxydans*, *Fructilactobacillus sanfranciscensis*, *Lactobacillus delbrueckii* subsp. *Bulgaricus*, and *Levilactobacillus brevis* ATCC 367.

Most of these horizontally transferred genes from *Lactobacillus* species were found in the microbiota of fermented vegetables. Among these 71 phage genes, four transposase genes (PP_00031, PP_01793, PP_02109, PP_02110) were associated with recombination, repair, and replication processes. Furthermore, the whole-genome-based transposase search identified members of transposases from IS1182, IS256, IS30, ISL3, IS3, and IS5 families using IS Finder, as summarized in Table S8.

### 3.3. Phenotypic Antibiotic Resistance and Safety-Related Gene Assessment

The susceptibility of JS21 to 14 antibiotics was assessed and antibiotic resistance genes (ARGs) were mined from the JS21 genome. JS21 exhibited resistance to vancomycin (30 μg), kanamycin (30 μg), sulfafurazole (300 μg), norfloxacin (10 μg), and ciprofloxacin (5 μg). Conversely, JS21 showed sensitivity to Penicillin G (10 U), Ampicillin (10 μg), Amoxicillin (20 μg), Cefotaxime Sodium (30 μg), Gentamicin (10 μg), Erythromycin (15 μg), Tetracycline (30 μg), and Minocycline (30 μg), with intermediate sensitivity to Enrofloxacin (10 μg).

Using ResFinder 4.3.1 for ARGs detection within the ResFinder and Disinfinder databases, a ClpL gene was identified, known to be ubiquitous in *L. plantarum* strains and confirmed via BLAST. Members of the ATP-dependent Clp protease family, including ClpL, are involved in degrading misfolded or damaged intracellular proteins, contributing to cellular resistance under high-temperature stress conditions [59,60]. Furthermore, the CARD database search yielded 195 genes with a concordance range of 21.9–75.8% and coverage of 9.5–100% for matched regions. The loose hits included genes associated with resistance mechanisms such as antibiotic target alteration (66), antibiotic target protection (13), antibiotic efflux (106), antibiotic inactivation (7), and antibiotic target replacement (3). No drug resistance genes were annotated when the identity was ≥90% and coverage was ≥60%. Since both databases primarily focus on the antibiotic resistance genes of pathogenic bacteria, ARGs of non-pathogenic bacteria such as *Lactobacillus* are typically not included. However, eleven ARGs in the JS21 genome were identified through a KEGG database search, including genes related to β-Lactams (8), Tetracyclines (1), Vancomycin (7), and Lincomycin (1) (refer to Table S9).

It is well-known that lactic acid bacteria, particularly the *Lactobacillus* species, exhibit a certain resistance profile to aminoglycoside [61,62,63]. In the genome of JS21, vancomycin resistance genes were identified, including phospho-N-acetylmuramoyl-pentapeptide-transferase (*mraY*, EC: 2.7.8.13), alanine racemase (*alr*, EC: 5.1.1.1), D-alanyl-D-alanine ligase (*ddl*, EC: 6.3.2.4), UDP-N-acetylmuramoyl-tripeptide-D-alanyl-D-alanine ligase (*murF*, EC: 6.3.2.10), UDP-N-acetylglucosamine–N-acetylmuramyl-(pentapeptide) pyrophosphoryl-undecaprenol N-acetylglucosamine transferase (*murG*, EC: 2.4.1.227), D-Ala-D-Ala carboxypeptidase (*vanY*, EC: 3.4.17.14), and D-Ala-D-Ala dipeptidase (*vanX*, EC: 3.4.13.22). Notably, *vanX* is highly specific for hydrolyzing D-Ala-D-Ala dipeptides, significant precursors of the cell wall [56,64]. Additionally, *tetM* (ribosomal protection tetracycline resistance protein, MFS transporter) and *lmrB* (DHA2 family, lincomycin resistance protein) were detected, which are related to tetracyclines and lincomycin. Major genes responsible for beta-lactam resistance, *pbp2A* (penicillin-binding protein 2A, EC: 2.4.1.129 3.4.16.4) and *penP* (beta-lactamase class A, EC: 3.5.2.6), were also identified in the JS21 genome. β-lactam-related transporter proteins, including *abcA*, *oppA*, *oppB*, *oppC*, *oppD*, and *oppF*, were detected. Despite the presence of these resistance genes, JS21 did not exhibit resistance to beta-lactams or tetracyclines, indicating the complex interplay between genotype and phenotype [65]. Phenotypically, JS21 displayed resistance to kanamycin, ciprofloxacin, norfloxacin, and sulfisoxazole, with moderate sensitivity to enrofloxacin, despite the absence of specific resistance genes. This phenomenon may be attributed to membrane impermeability and/or multidrug efflux transporters, as evidenced by the presence of *efrA*, *efrB*, *mdlA*, *mdlB*, *patA*, and *patB* (confirmed in Tables S10 and S12), contributing to reduced susceptibility to these drugs.

Lactic acid bacteria are commonly found in natural foods and the human intestine, and they are frequently utilized as major ingredients in probiotic products due to their numerous beneficial properties. However, concerns arise regarding their resistance mechanisms, particularly their intrinsic resistance to antibiotics like vancomycin, metronidazole, and colistin, which may confer a survival advantage in the gut when administered [66]. Pathogenic bacteria can acquire resistance through plasmids or mutations, and they may obtain resistance genes from the surrounding environment. Documented cases of transferable drug resistance underscore the need for stringent monitoring of the safety profile of probiotic microbes [67,68]. Before approving certain bacterial strains as probiotics, breakpoints for interpreting drug resistance should be carefully evaluated. In the Protein BLAST screening conducted in this study for antibiotic resistance genes, no evidence of horizontal gene transfer was found (refer to Table S10).

According to the VFDB database settings, 274 potential virulence factors were annotated in JS21. However, the similarities between these genes and those in the VFDB database were mostly below 60%. Conversely, based on the annotations from the KEGG and COG databases, most of the annotated potential virulence genes were involved in the biosynthetic metabolism of secondary metabolites, biofilm formation, starch and sucrose metabolism, and the biosynthesis of cofactors. Therefore, JS21 does not appear to produce secondary harmful metabolites with toxic effects. The annotation results from different databases suggest that these genes may not be virulence genes. For example, genes associated with environmental tolerance may be considered virulence factors for pathogenic bacteria. However, for probiotics such as lactobacilli, these genes are involved in the survival of the strain in the gut and are no longer virulence genes for probiotics.

### 3.4. Carbohydrate Fermentation Patterns and Active Enzymes

The association between genotype and phenotype facilitated the identification of genes responsible for transporting and metabolizing specific carbon sources. Functional annotation of these orthogroups provided insights into the metabolic pathways underlying the fermentative capabilities of selected LAB strains. According to the API 50 CHL test results, JS21 could metabolize 23 different carbohydrates out of 49 tested (Table 2). Y44 and ATCC14917 strains were previously reported by Gao et al. (2020) [47]. In contrast to ATCC 14917 and Y44, JS21 was incapable of metabolizing L-arabinose, D-raffinose, D-turanose, and gluconates. Notably, although JS21 possessed genes related to gluconate metabolism, such as gluconate transport protein (K03299), decarboxylating 6-phosphogluconate dehydrogenase (*gntZ*, K00033), and 2,5-didehydro-D-gluconate-like protein (*ydhP*, K19577), it could not metabolize gluconates. This discrepancy may be attributed to the lack of expression of the aforementioned genes encoding the specific enzymes required for gluconate metabolism [69]. Furthermore, JS21 exhibited metabolic capacity for inositol and glycerol. Previous studies have demonstrated that myo-inositol and glycerol serve as fermentable energy sources utilized by *L. plantarum* in the distal gut [70]. These differences in metabolic capabilities may arise from the physiological and genetic adaptations of the strains to their respective ecological niches.

The phosphoenolpyruvate-dependent sugar phosphotransferase system (PTS) in lactic acid bacteria is an important transporter of carbohydrates [71]. In the JS21 strain, the entire PTS enzyme system is encoded by its genome, comprising PTS system enzyme I (general enzyme gene, *ptsI*, K08483), phosphocarrier protein HPr gene (*ptsH*, K02784), 1-phosphofructokinase (*fruK*, K00882), and 53 complete or incomplete substrate-specific enzyme II (EII) complex genes (refer to Table S13). These complex genes appear to be specific to various substrates such as cellobiose, fructose, mannose, mannitol, galactosamine, N-acetylglucosamine, sucrose, beta-glucoside, and ascorbate, although their exact substrate specificities remain unidentified. It is widely known that various sugar transporters have the ability to transport more than one substrate [72]. Additionally, several carbohydrate-specific common ABC transporters are also present, as listed in Table S12.

Genes encoding enzymes associated with both intact Embden–Meyerhof–Parnas (EMP) pathways (Figure S2) and phosphoketolase (PK) (Figure S3) pathways were predicted in the genome of JS21 strains, indicating that JS21 is facultative heterofermentative *Lactobacillus* species. Glucose is converted into fructose-1,6-bisphosphate (FBP) by glucokinase [EC: 2.7.1.2], glucose-6-phosphate isomerase [EC: 5.3.1.9], and 6-phosphofructokinase 1 [EC: 2.7.1.11]. FBP undergoes further metabolism by fructose-bisphosphate aldolase, class II [EC: 4.1.2.13], glyceraldehyde 3-phosphate dehydrogenase [EC: 1.2.1.12], phosphoglycerate kinase [EC: 2.7.2.3], 2,3-bisphosphoglycerate-dependent phosphoglycerate mutase [EC: 5.4.2.11], and enolase [EC: 4.2.1.11] to produce phosphoenolpyruvate. Finally, pyruvate is produced for entry into the tricarboxylic acid cycle (TCA cycle) or to generate lactic acid [73]. Additionally, the *fruK* gene encoding 1-phosphofructokinase [EC: 2.7.1.56] was detected, which is crucial for differentiating hetero- and homofermentative lactobacilli species [74]. Homofermentative species encode 1-phosphofructokinase for the degradation of D-mannose, which is lacking in obligately heterofermentative species [75]. It can be inferred that JS21 possesses a facultative heterofermentative carbohydrate metabolism similar to other *L. plantarum* strains. However, the JS21 genome does not encode all TCA cycle-related proteins, as observed in prevalent *L. plantarum* strains [43,47,55].

The JS21 bacteria could produce both forms of lactate and contained six copies of L-lactate dehydrogenase (*ldh*) and two copies of D-lactate dehydrogenase (*ldhA*). Additionally, JS21 had two copies of alcohol dehydrogenase (*adh*) genes. The type of product formed by *adh* varied depending on the substrate. It generated aldehydes when primary alcohols were used, and ketones when secondary alcohols were the substrates [76]. Generally, aldehydes and ketones contribute to the distinctive flavors of fermented foods [77]. The impact of these reaction products on the specific flavor of Jiangshui was significant.

The API results showed that JS21 could metabolize both glucose and lactose. JS21 possessed three copies of UDP-glucose 4-epimerase (*galE*), three copies of aldose 1-epimerase (*galM*), one copy of UDP-glucose-hexose-1-phosphate uridylyl transferase (*galT*), one copy of galactokinase (*galK*), and two copies of the beta-galactosidase (*bgaB*) gene. All enzymes necessary for the Leloir metabolic pathway were present. In this pathway, lactose was converted into D-glucose by beta-galactosidase [EC: 3.2.1.23], aldose 1-epimerase [EC: 5.1.3.3], galactokinase [EC: 2.7.1.6], UDP-glucose-hexose-1-phosphate uridylyl transferase [EC: 2.7.7.12], and phosphoglucomutase [EC: 5.4.2.2], before entering the glycolysis pathway.

According to KEGG mapper results, JS21 possesses a total of 203 genes related to carbohydrate metabolism encoded in its genome (Table S11). These genes are distributed across various pathways as follows: twenty-three genes associated with glycolysis/gluconeogenesis, seven genes linked to the TCA cycle, fifteen genes involved in the pentose phosphate pathway, four genes related to pentose and glucuronate interconversions, twenty-one genes associated with fructose and mannose metabolism, eighteen genes involved in galactose metabolism, three genes related to ascorbate and aldarate metabolism, twenty-three genes associated with starch and sucrose metabolism, twenty-seven genes involved in amino sugar and nucleotide sugar metabolism, twenty-five genes related to pyruvate metabolism, eleven genes involved in glyoxylate and dicarboxylate metabolism, ten genes linked to propanoate metabolism, two genes related to C5-branched dibasic acid metabolism, six genes involved in inositol phosphate metabolism, and eight genes linked to butanoate metabolism. Additionally, JS21’s genome encodes a variety of CAZymes, including 32 glycosyl transferases, 48 glycoside hydrolases, two carbohydrate-binding modules, 11 auxiliary activity enzymes, and 16 carbohydrate esterases. However, no polysaccharide lyases were identified in the CAZy annotation results. The results showed that JS21 had good carbohydrate utilization potential and specific adaptability in the gastrointestinal tract.

### 3.5. Secondary Metabolite Biosynthetic Gene Clusters

Secondary metabolites play important roles because of their antibacterial, antiviral, and anticancer activities.. Genome mining has become a widely used method for analyzing biosynthetic gene clusters (BGCs) of these compounds [31,78]. Four BGCs were predicted in the JS21 genome, including ribosomally synthesized and post-translationally modified peptides (RiPP-like), type III polyketide synthases (T3PKS), terpenes, and cyclic lactone autoinducer (Table S14).

**Figure 5:**
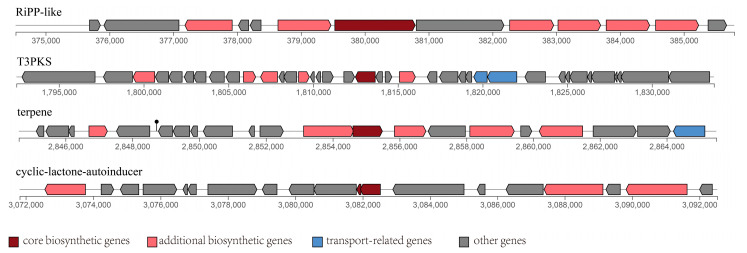
Predicted biosynthetic gene clusters encoding secondary metabolites in the *Lactiplantibacillus plantarum* JS21 genome.

A cluster of 13 genes for bacteriocin biosynthesis with a total length of approximately 11.2 kb was identified by the antiSMASH database (Figure 5). This gene cluster encoded transport-related genes and plantaricin precursor genes (*plnE*, *plnF*), all of which were confirmed by protein BLAST (Table S15). Choi et al. [79] conducted a comparative genomic analysis of 54 complete genome sequences and found that the *plnE*/*F* locus was commonly present in *L. plantarum* strains isolated from various ecological niches. The genes associated with the biosynthesis of *plnE*/*F* were conserved throughout *L. plantarum*. Both plantaricins formed pores in the plasma membrane of target bacteria, exhibiting complementary ion selectivity [80]. Each plantaricin contained multiple transmembrane structural domains contributing to immunological activity [81]. Both *plnE* and *plnF* in JS21 contained “GxxxG” motifs. Fimland et al. [82] proposed that *plnE*/*F* interacted with the G_30_xxxG_34_ motif in *plnF* via the G_5_xxxG_9_ or G_20_xxxG_24_ motifs in *plnE*. Ekblad et al. [83] further determined that the G5xxxG9 motif in *plnE* interacted with the G_30_xxxG_34_ motif in *plnF* in an antiparallel manner through mutant analysis, and the mutation of the “GxxxG” motif in *plnE/F* did not significantly affect bacteriocin activity. Besides the core genes, the presence of the HlyD family secretion protein, the bacteriocin ABC-transporter ATP-binding and permease component, the transport/processing ATP-binding protein ComA, and the CPBP family intramembrane metalloprotease were verified in the plantaricin gene cluster. Additionally, the genes encoding precursor peptides and bacteriocin immunity proteins were usually located back-to-back in the same operon to protect the strain from its own bacteriocins. However, the bacteriocin immunity proteins in JS21 were found in the terpene synthesis gene cluster.

Type III polyketide synthases (T3PKS) identified in the JS21 genome play a crucial role in producing bacteriocins, contributing to food safety. T3PKS are among the most abundant BGCs in all LAB genera [42]. The core biosynthetic gene of T3PKS, *mvaS*, encodes hydroxymethylglutaryl-CoA synthase [EC: 2.3.3.10], which is sensitive to feedback inhibition by acetoacetyl-CoA. Hydroxymethylglutaryl-CoA, a metabolite from fatty acid catabolism, has the potential to inhibit the growth of pathogenic microorganisms during food fermentation [84]. T3PKS contains separate proteins equivalent to the functional domains of type I and type II PKS (ketosynthase, KS). Using acyl-CoA as a substrate, T3PKS iteratively synthesizes polyketones with various chain lengths [85]. The JS21 genome also encodes thioesterase (gene0554), acyl-CoA thioesterase (gene1430), acyl carrier protein (gene1381, gene1417), and 1-acyl-sn-glycerol-3-phosphate acyltransferase (Table S16).

Terpene synthesis-related genes (Table S17) are annotated in the JS21 genome by KEGG and antiSMASH databases. These include hydroxymethylglutaryl-CoA synthase [EC: 2.3.3.10], hydroxymethylglutaryl-CoA reductase [EC: 1.1.1.88], phosphomevalonate kinase [EC: 2.7.4.2], mevalonate kinase [EC: 2.7.1.36], diphosphomevalonate decarboxylase [EC: 4.1.1.33], and isopentenyl-diphosphate Delta-isomerase [EC: 5.3.3.2]. Δ^3^-isopentenyl pyrophosphate and Δ^2^-isopentenyl pyrophosphate, synthesized via the mevalonate pathway, serve as isopentenyl donors from primary metabolism and can be used as synthetic building blocks for terpene secondary metabolites [86]. The gene cluster also encodes the phytoene desaturase family protein and the phytoene synthase family protein associated with carotenoid production. The cyclic lactone autoinducer peptide is a core gene in the cyclic lactone autoinducer gene cluster (Table S18). Mull et al. [87] discussed that cyclic peptides, similar to cyclic lactone autoinducer peptide, regulate critical pathways of signal transduction and target polysaccharide biosynthesis and sugar utilization enzymes.

### 3.6. Probiotic Properties

Probiotic characterization tests were conducted to confirm the presence or absence of features essential for probiotic functionality. β-hemolysis, a crucial indicator of strain safety, was assessed, revealing that JS21 exhibited no β-hemolytic activity. Colonization ability on the intestinal wall is a desirable property of probiotic bacteria [88]. Bacterial adhesion ability is dependent on the cell surface hydrophobicity and auto-aggregation capacity, which is the key to probiotic colonization in the animal intestine. In addition, co-aggregation eliminates gastrointestinal pathogen colonization by preventing pathogens from attaching to host tissues. [14,88]. JS21 demonstrated a cell surface hydrophobicity of 60.04 ± 0.96% and an auto-aggregation capacity of 42.63 ± 1.06%. Co-aggregation rates of JS21 with target pathogens, including *E. coli* (ATCC 25922), *E. coli* (K88), *S. aureus* (CMCC 26003), and *L. monocytogenes* (CICC 21635), were 31.69 ± 0.51%, 30.97 ± 0.83%, 44.92 ± 0.30%, and 35.35 ± 0.70%, respectively. JS21 exhibited significant aggregation ability with *S. aureus*.

Intestinal colonization ability is an important indicator for screening probiotics, which is related to the structure and properties of the cell surface of the strain [89]. Liu et al. [38] isolated 15 strains of *L. plantarum* from fermented vegetables in Shaanxi, 12 of which exhibited auto-aggregation rates higher than 60%. Previous studies have reported that certain lactobacilli strains, such as *Lb. acidophilus* and *L. johnsonii*, displayed surface hydrophobicity as low as 2–5%, while the surface hydrophobicity of *L. plantarum* DY46 was only 4.38%, significantly lower than that of JS21 [27,90,91]. Bacterial cell wall components and other structures, including mucus-binding proteins, adhesins, surface layer proteins, fibronectin-binding proteins, exopolysaccharides, and lipoteichoic acids, confer an advantage to bacteria for colonization and adhesion to host epithelial cells [92,93,94]. In this study, the presence of some of these structures was confirmed in the genome of JS21 and they are summarized in Table S19.

Studies have indicated that a minimum of 10^6^–10^7^ CFU/g of lactic acid bacteria is required to colonize the intestinal tract and exert probiotic effects on the human body [95]. However, when active lactic acid bacteria are ingested, they experience a significant loss of activity due to exposure to low pH in gastric acid and high bile salts in the intestinal fluid. For lactic acid bacteria to fulfill their probiotic role in the gut, they must withstand the gastrointestinal tract environment and survive, necessitating tolerance to strong acids and high bile acids [96,97,98]. The viable counts of JS21 significantly decreased from 10^9^ CFU/mL to 10^4^ CFU/mL after incubation with 0.3% bovine bile salts for 2 h, indicating poor tolerance to bile salts. However, JS21 exhibited a certain degree of tolerance to the low pH environment of the intestinal tract. The main component of gastric acid is hydrochloric acid, typically with a pH of 3.0, dropping to around 2.0 or even lower during fasting. JS21’s survival rate after gastric fluid treatment (at pH 2.0) was 18.91%, but the bacteria demonstrated a proliferative state after incubation in intestinal fluid. In contrast, the survival rate of JS21 after gastric fluid treatment (at pH 3.0) was 143.75%, indicating a significant proliferative tendency (Table S21). Bile salt hydrolase genes associated with bile salt tolerance were not annotated in the JS21 genome, correlating with the phenotype of intolerance to bile salts. The resistance of JS21 to acidic environmental stress in the gastrointestinal tract was associated with stress resistance genes, DNA and protein protection, and repair DNA-related genes (Table S19). Notably, cation–proton antiporters (*nhaK*, *ctpE*), ABC ATPases, F_0_F_1_-type ATP synthase, and chaperones (Clp ATPases) played roles in homeostasis and intracellular pH regulation, contributing to acid and bile resistance. Similar acid resistance mechanisms have been reported in *L. plantarum* Y44 [47], *W. cibaria* CH2 [99], and *L. fermentum* YLF016 [100].

**Figure 6:**
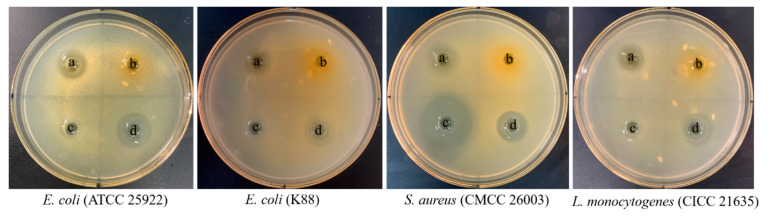
The inhibition zone diameters of JS21 against *E. coli* ATCC 25922, *E. coli* K88, *S. aureus* CMCC 26003 and *L. monocytogenes* CICC 21635. a—CFS; b—NC; c—Ampicillin; d—Kanamycin.

Pathogenic bacteria contribute to intestinal diseases, with traditional treatment primarily relying on antibiotics. However, excessive antibiotic use can disrupt the structure of the intestinal microbiome and lead to drug resistance in pathogenic bacteria [101]. Lactic acid bacteria inhibit intestinal pathogenic bacteria by producing various metabolites, including short-chain fatty acids, lactic acid, formic acid, bacteriocins, hydrogen peroxide, and small molecule peptides [102]. According to antimicrobial activity test results, the cell-free supernatant (CFS) of JS21 significantly inhibited the growth of *E. coli* (K88), *E. coli* (ATCC 25922), *S. aureus* (CMCC 26003), and *L. monocytogenes* (CICC 21635) (Figure 6, Table S22), while delaying the entry of pathogens into the logarithmic growth phase (Figure S5). Compared with ampicillin, the antimicrobial ability of CFS of JS21 against *S. aureus* was lower, whereas it was higher against the other three strains. In addition, the inhibition zone sizes of CFS were close to those of kanamycin. It was suggested that the CFS of JS21 had comparable antibacterial activity to the used standard antibiotics.

The JS21 genome was annotated with bacteriocin *plnE/F*-encoding genes, multiple lactate dehydrogenase genes, and extracellular polysaccharide-related genes (Table S19). During growth, the JS21 strain reduced the pH of the medium to 3.74 (Figure S6). Probiotic bacteria typically resist pathogenic microorganisms by producing organic acids [103]. Extracellular polysaccharides (EPS) effectively inhibit the biofilm formation of pathogenic bacteria, thereby affecting their viability [104]. EPS (32 kDa) synthesized by *L. plantarum* YW32 exhibited a dose-dependent inhibitory effect on biofilm formation in a wide range of pathogenic bacteria (*Staphylococcus aureus*, *Listeria monocytogenes*, *Pseudomonas aeruginosa*, and *Salmonella enterica* subsp. *enterica* serovar Typhi) [105]. This inhibitory mechanism may involve EPS interfering with biofilm activity by modifying bacterial cell surfaces, hindering the initial attachment of pathogenic bacteriophages, and down-regulating the expression of genes involved in biofilm formation via signal molecules [106].

Additionally, EPS from *L. plantarum* exhibited antibacterial effects. For instance, biosurfactants composed of protein and polysaccharide fractions from *L. plantarum* CFR 2194 demonstrated significant antibacterial activity against various foodborne pathogens, attributed to structural disruption of the bacterial cell envelope, particularly the peptidoglycan layer, by the EPS [107,108]. The protease S-ribosylhomocysteine lyase (LuxS), a key enzyme for synthesizing population-sensing AI-2 signal molecules, was identified in JS21. AI-2 plays a crucial role in microbial intraspecies and interspecies communication, with LuxS genes present in over 80 bacterial species [109,110]. Many bacterial physiological phenomena are closely linked to the LuxS-mediated group sensing system, which regulates bacterial activity [111].

Moreover, both the chromosome of JS21 and the plasmid pWS05 encoded genes related to biofilm formation, listed in Table S19. Costerton et al. [112] identified biofilms as closed matrices produced by bacterial populations for adhesion to interfaces, facilitating bacterial adhesion and community coexistence. This matrix comprises extracellular polymers secreted by the bacteria themselves, primarily consisting of EPS, proteins, and extracellular DNA. Biofilms can form at any interface [113,114]. Probiotic biofilms aid in survival and colonization *in vivo*, enhancing resistance to pathogenic bacteria.

## 4. Conclusions

Genomic analysis, physiological assessment, and validation of probiotic functions were conducted on *L. plantarum* JS21, isolated from Jiangshui, to determine its probiotic properties. This study briefly discussed its genomic characteristics, carbohydrate fermentation pattern, secondary metabolite biosynthesis, antibiotic resistance status, prophage presence, and associated horizontal gene transfer. Genomic analysis revealed that JS21 follows a facultative heterofermentative sugar metabolism, where hexoses are metabolized via glycolysis and pentoses via the pentose phosphate pathway. Furthermore, JS21 was predicted to synthesize *plnE* and *plnF* peptides, polyketides, and terpenoids, associated with its ability to inhibit pathogenic bacteria. Its strong acid-producing capacity also contributed to its inhibition ability. The genome of JS21 harbored five prophage regions—three complete and two incomplete—and seventy-one genes were identified as horizontally transferred from different bacteria, including four transposase genes. However, all detected resistance genes in JS21 were intrinsically originated, with no horizontal gene transfer observed. JS21 exhibited high surface hydrophobicity and tolerance to the acidic environment of the simulated human gastrointestinal tract, attributed to the presence of Na^+^/H^+^ antiporters, F_0_F_1_ ATPase, and other resistance genes. Overall, *L. plantarum* JS21 shows promising probiotic characteristics confirmed by *in vitro* analysis and may serve as a potential candidate for dietary supplementation. However, further *in vivo* experiments are necessary to elucidate its functional characteristics comprehensively.

## Figures and Tables

**Table 1:** Genomic properties of *Lactiplantibacillus plantarum* JS21.

Type	Name	Size (bp)	G+C (%)	Genes	Pseudogenes (Total)	Protein	rRNA	tRNA	Non-Coding RNA	CRISPR_Array	CRISPR_Repeat	CRISPR_Spacer
Chromosome	-	3.126.700	44.79	3090	223	2747	64	16	40	6	85	79
Plasmid	pWS01	72.513	42.33	79	12	67	-	-	-	-	-	-
Plasmid	pWS02	71.633	40.97	71	6	65	-	-	-	-	-	-
Plasmid	pWS03	54.200	40.14	55	10	45	-	-	-	-	-	-
Plasmid	pWS04	51.130	41.74	70	5	65	-	-	-	-	-	-
Plasmid	pWS05	47.792	43.9	42	8	34	-	-	-	-	-	-

**Table 2:** Carbohydrate fermentation profile of *Lactoplantibacillus plantarum* JS21.

Sugars	JS21
Control	−
Glycerol	+
Erythritol	−
D-Arabinose	−
L-Arabinose	−
D-Ribose	+
D-Xylose	−
L-Xylose	−
Adonitol	−
Methyl-β-D-xylopyranoside	−
D-Galactose	+
D-Glucose	+
D-Fructose	+
D-Mannose	+
D-Sorbose	−
D-Rhamnose	−
Dulcitol	−
Inositol	+
D-Mannitol	+
D-Sorbitol	+
Methyl-α-D-mannopyranoside	+
Methyl-α-D-glucopyranoside	−
N-Acetylglucosamine	+
Amygdalin	+
Arbutin	+
Esculin ferric citrate	+
Salicin	+
D-Cellobiose	+
D-Maltose	+
D-Lactose	+
D-Melibiose	+
D-Sucrose	+
D-Trehalose	+
Inulin	−
D-Melezitose	+
D-Raffinose	−
Amidon (Starch)	−
Glycogen	−
Xylitol	−
Gentiobiose	+
D-Turanose	−
D-Lyxose	−
D-Tagatose	−
D-Fucose	−
L-Fucose	−
D-Arabitol	−
L-Arabitol	−
Gluconate	−
2-Keto-gluconate	−
5-Keto-gluconate	−

Notes: (+) positive, (−) negative.

## Data Availability

The original contributions presented in the study are included in the article/Supplementary Materials; further inquiries can be directed to the corresponding author. NGNC repository access number GWHEROS00000000.

## Supplementary Materials

The following are available online at: https://www.mdpi.com/article/10.3390/foods13071082/s1, Figure S1: Location of phage on the JS21 genome; Figure S2: Annotated map of glycolysis/gluconeogenesis pathways in JS21; Figure S3: Annotated map of pentose phosphate (phospoketolase) pathways in JS21; Figure S4: The graphical presentation of galactose metabolism pathway of *Lactiplantibacillus plantarum* JS21 was obtained from KEGG Mapper; Figure S5: The ability of CFS to inhibit the growth of pathogenic bacteria; Figure S6: Growth curve of JS21 versus acid production rate curve. Table S1: The predicted prophage regions; Table S2: The first prophage (intact) region elements; Table S3: The second prophage (intact) region elements; Table S4: The third prophage (intact) region elements; Table S5: The fourth prophage (incomplete) region elements; Table S6: The fifth prophage (incomplete) region elements; Table S7: Horizontal gene transfer of prophage region proteins using ProteinBLAST; Table S8: The predicted transposases of the JS21 genome by using IS Finder; Table S9: Match between antibiotic resistance gene search using KofamKOALA (KEGG Orthology) web servers and phenotypic antibiotic resistance results; Table S10: Horizontal gene transfer screening for antibiotic resistance genes; Table S11: Carbohydrate metabolism genes annotated by KEGG orthology; Table S12: KEGG (BlastKOALA) orthology search results for ABC transporters; Table S13: Phosphotransferase system (PTS) annotated by KEGG (BlastKOALA); Table S14: The predicted biosynthetic gene clusters of secondary metabolites; Table S15: The RiPP-like region elements; Table S16: The T3PKS region elements; Table S17: The terpene region elements; Table S18: The cyclic-lactone-autoinducer region elements; Table S19: Putative probiotic genes are found in the genome; Table S20: The hydrophobicity, auto-aggregation, and co-aggregation; Table S21: Tolerance of JS21 to simulated gastrointestinal fluids; Table S22: JS21 Inhibition zone results of CFS against pathogens’ bacteria.
