# Supplementary material for: Characterization of Genomic, Physiological, and Probiotic Features of Lactiplantibacillus plantarum JS21 Strain Isolated from Traditional Fermented Jiangshui
Source: Foods. 2024 Apr 1;13(7):1082. doi: 10.3390/foods13071082 (PMC11011416; doi:10.3390/foods13071082)
Supplement: Supplementary file 1 [file foods-13-01082-s001.zip › foods-2923675-supplementary.pdf]

Characterization of Genomic, Physiological, and Probiotic Features *Lactiplantibacillus plantarum* JS21 Strain Isolated from of Traditional Fermented "Jiangshui"

Yang Liu[1,†], Shanshan Wang[1,2,*,†], Ling Wang[1,3,4], Hongzhao Lu[1,3,4], Tao Zhang [1,5,6], Wenxian Zeng [1,3,4]

1. School of Biological Science and Engineering, Shaanxi University of Technology, Hanzhong 723001, China;
2. QinLing-Bashan Mountains Bioresources Comprehensive Development C. I. C., Hanzhong 723001, China;
3. Engineering Research Center of Quality Improvement and Safety Control of Qinba Special Meat Products, Hanzhong 723001, China;
4. Shaanxi Union Research Center of University and Enterprise for Zhenba Bacon, Hanzhong 723001, China;
5. Qinba State Key Laboratory of Biological Resources and Ecological Environment, Hanzhong 723001, China;
6. Shaanxi Province Key Laboratory of Bio-resources, Hanzhong 723001, China

[*] CONTACT: Shanshan WANG, School of Biological Science and Engineering, Shaanxi University of Technology, No. 1 Dongyihuan Road, Hanzhong, Shaanxi 723000, China. Email: wss@snut.edu.cn.

†These authors contributed equally to this work.

Supplementary data

Figure S1. Location of phage on the JS21 genome.

Figure S2. Annotated map of glycolysis/gluconeogenesis pathways in JS21.

Figure S3. Annotated map of pentose phosphate (phospoketolase) pathways in JS21.

Figure S4. The graphical presentation of galactose metabolism pathway of *Lactiplantibacillus plantarum* JS21 was obtained from KEGG Mapper.

Figure S5. The ability of CFS to inhibit the growth of pathogenic bacteria.

Figure S6. Growth curve of JS21 versus acid production rate curve.

Table S1. The predicted prophage regions.

Table S2. The first prophage (intact) region elements.

Table S3. The second prophage (intact) region elements.

Table S4. The third prophage (intact) region elements.

Table S5. The fourth prophage (incomplete) region elements.

Table S6. The fifth prophage (incomplete) region elements.

Table S7. Horizontal gene transfer of prophage region proteins using ProteinBLAST.

Table S8. The predicted transposases of the JS21 genome by using IS Finder.

TableS9. Match between antibiotic resistance gene search using KofamKOALA (KEGG Orthology) web servers and phenotypic antibiotic resistance results.

Table S10. Horizontal gene transfer screening for antibiotic resistance genes.

Table S11. Carbohydrate metabolism genes annotated by KEGG orthology.

Table S12. KEGG (BlastKOALA) orthology search results for ABC transporters.

Table S13. Phosphotransferase system (PTS) annotated by KEGG (BlastKOALA).

Table S14. The predicted biosynthetic gene clusters of secondary metabolites.

Table S15. The RiPP-like region elements.

Table S16. The T3PKS region elements.

Table S17. The terpene region elements.

Table S18. The cyclic-lactone-autoinducer region elements.

Table S19. Putative probiotic genes are found in the genome.

Table S20. The hydrophobicity, auto-aggregation, and co-aggregation.

Table S21. Tolerance of JS21 to simulated gastrointestinal fluids.

Table S22. JS21 Inhibition zone results of CFS against pathogens bacteria.

Figure S1. Location of phage on the JS21 genome (green marked regions: intact and the score >90, blue marked regions questionable and score between 70 and 90, the red-coloured regions: incomplete and the score <70)

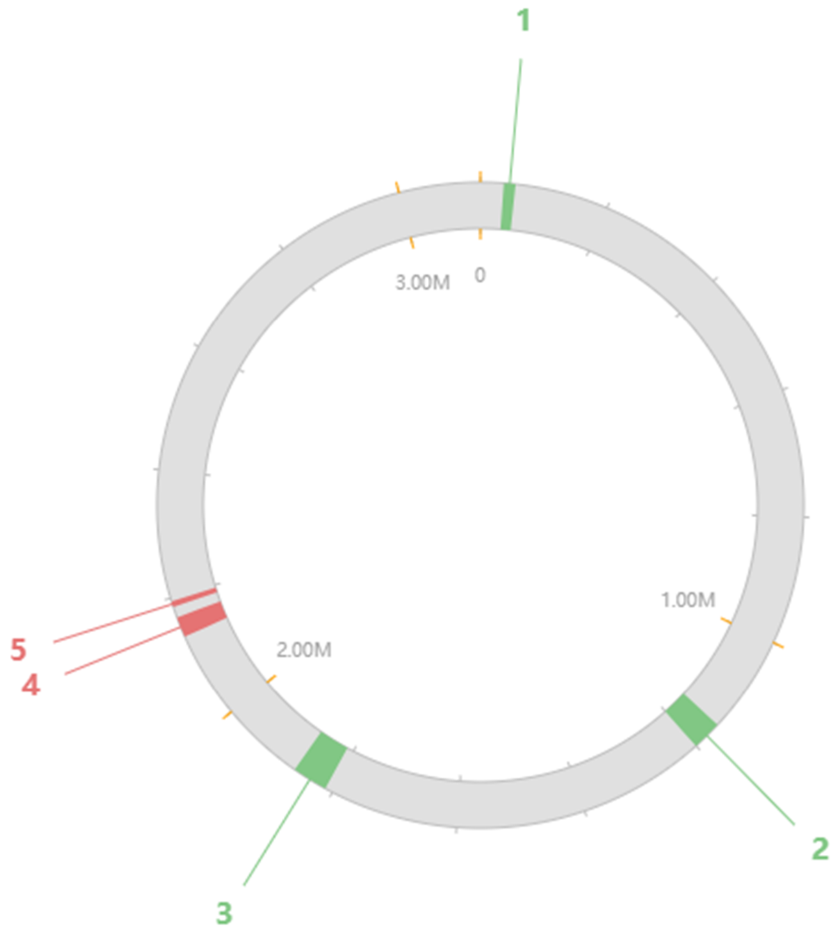

Figure S2. Annotated map of **glycolysis/gluconeogenesis** pathways in JS21 (Green coloured EC numbers indicate the presence of the pathway enzymes)

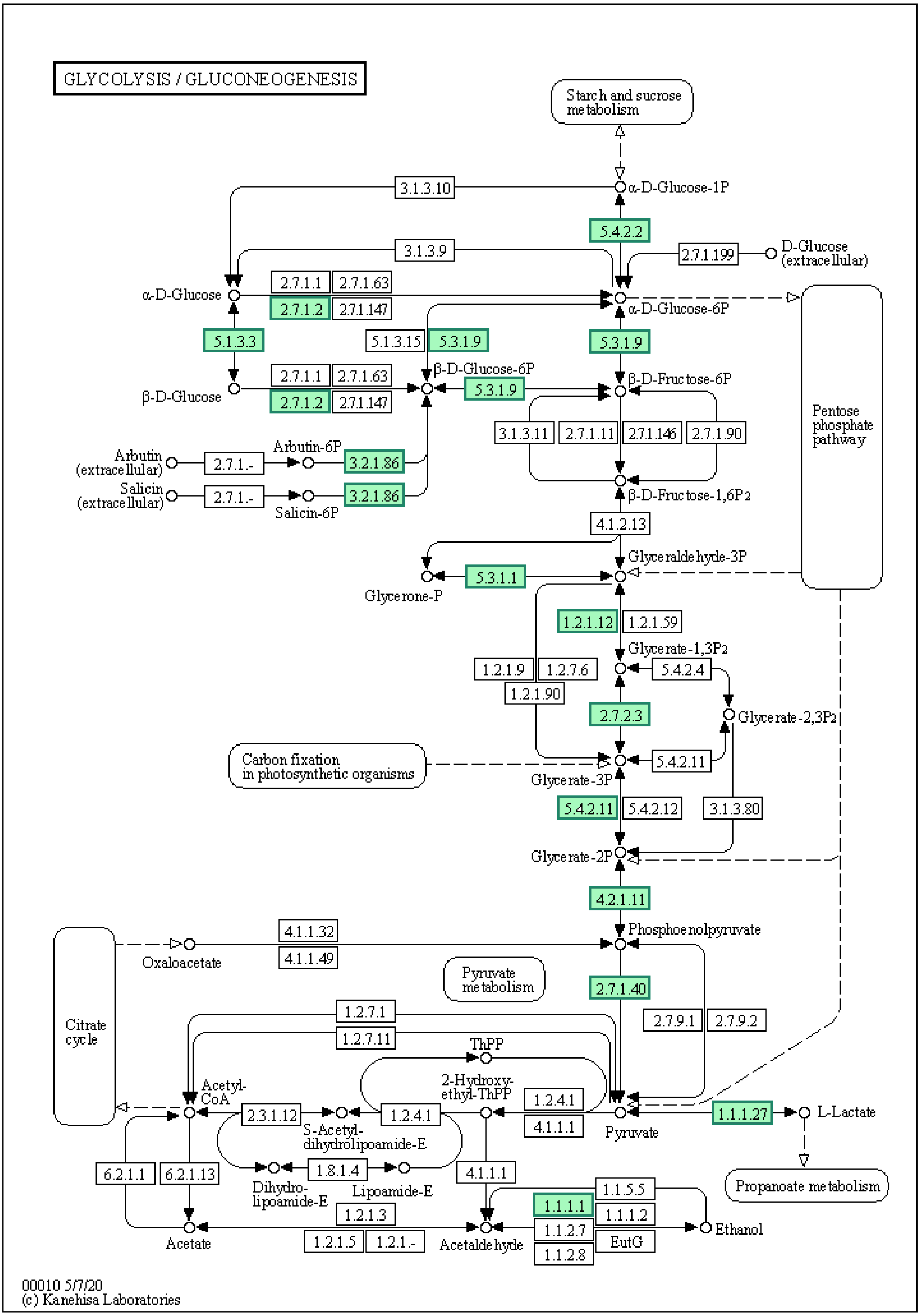

Figure S3. Annotated map of pentose phosphate (phospoketolase) pathways in JS21 (Green coloured EC number indicates the presence of the pathway enzymes).

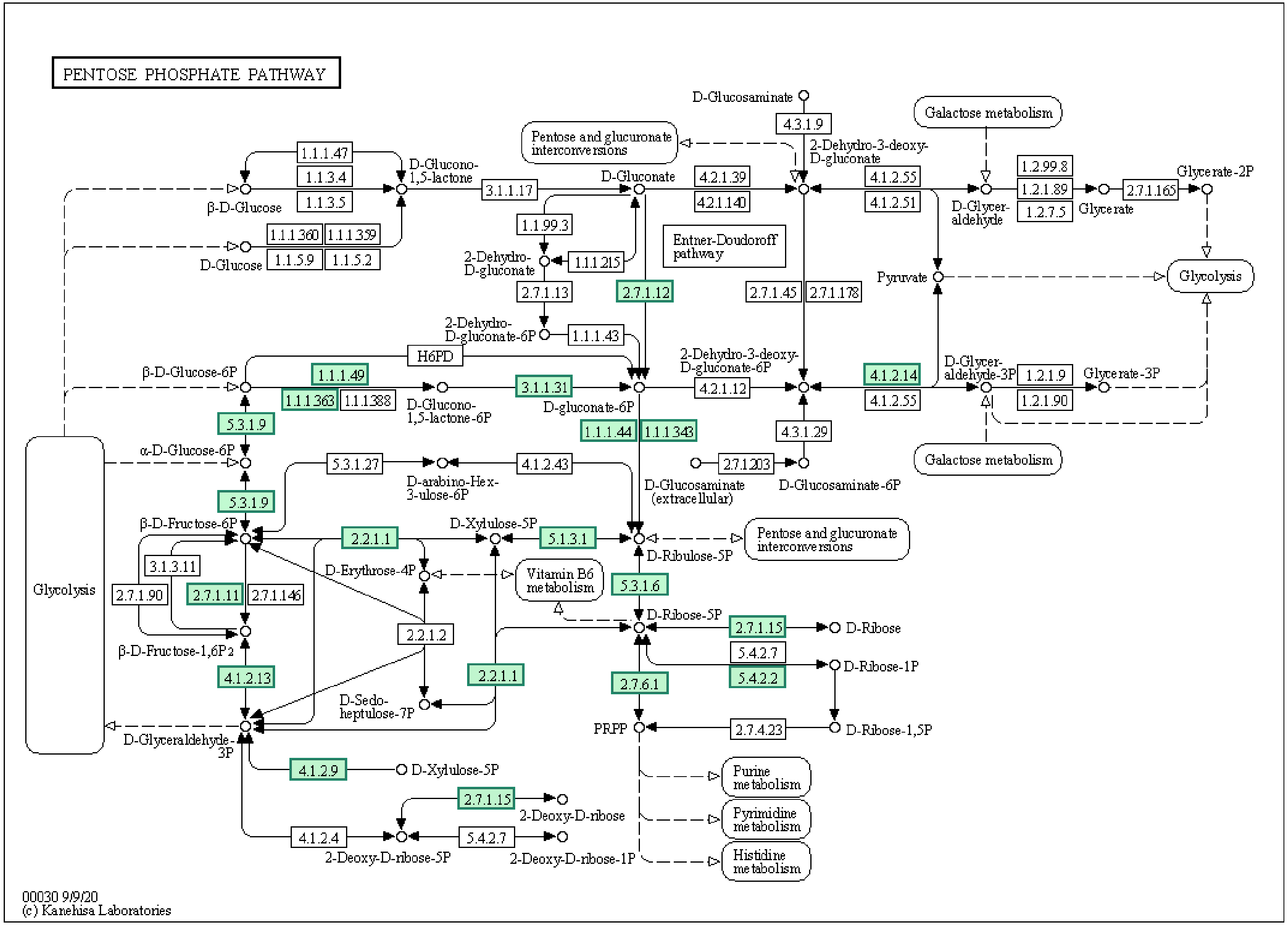

Figure S4. The graphical presentation of galactose metabolism pathway of *Lactiplantibacillus plantarum* JS21 was obtained from KEGG Mapper (Green coloured EC number indicates the presence of the pathway enzymes).

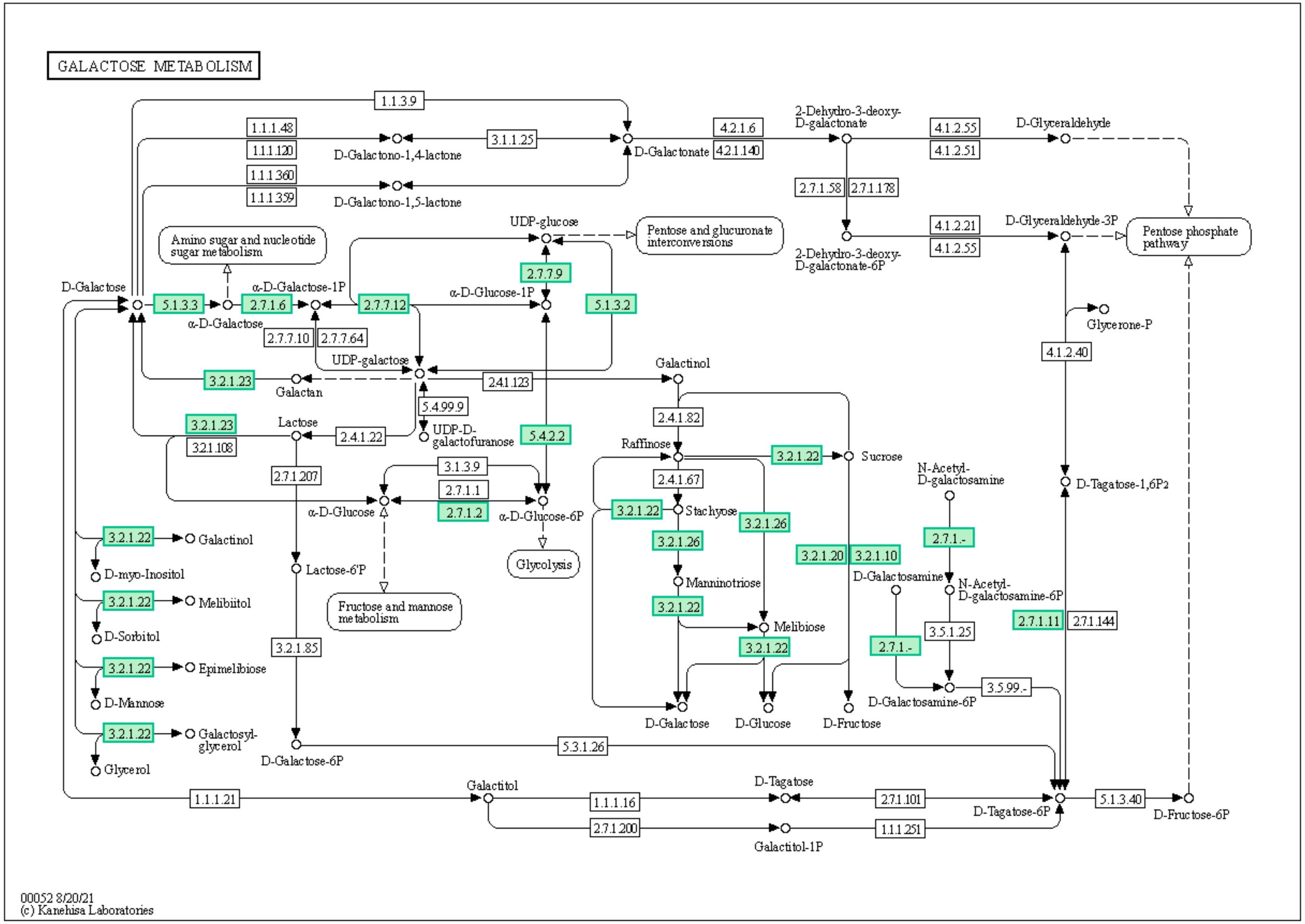

Figure S5. The ability of CFS to inhibit the growth of pathogenic bacteria. a- *Escherichia coli* ATCC 25922, b- *Escherichia coli* K88, c- *Staphylococcus aureus* CMCC 26003, d- *Listeria monocytogenes* CICC 21635.

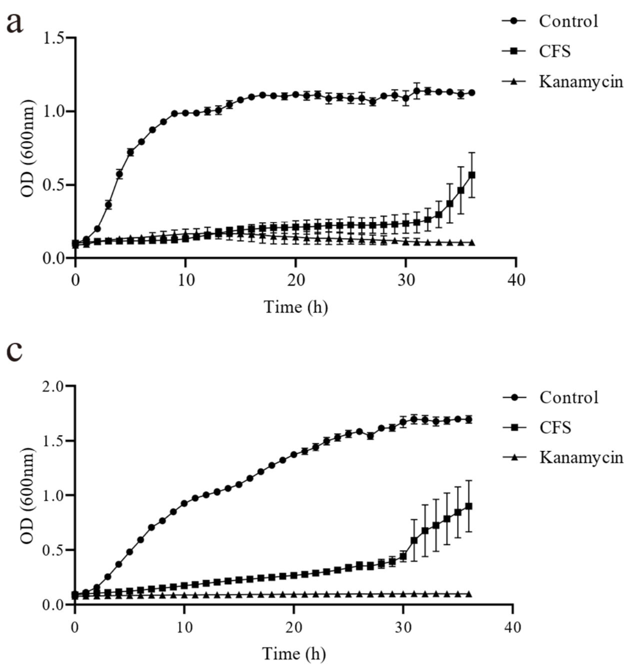

Figure S6. Growth curve(a) of JS21 versus acid production rate curve (b)

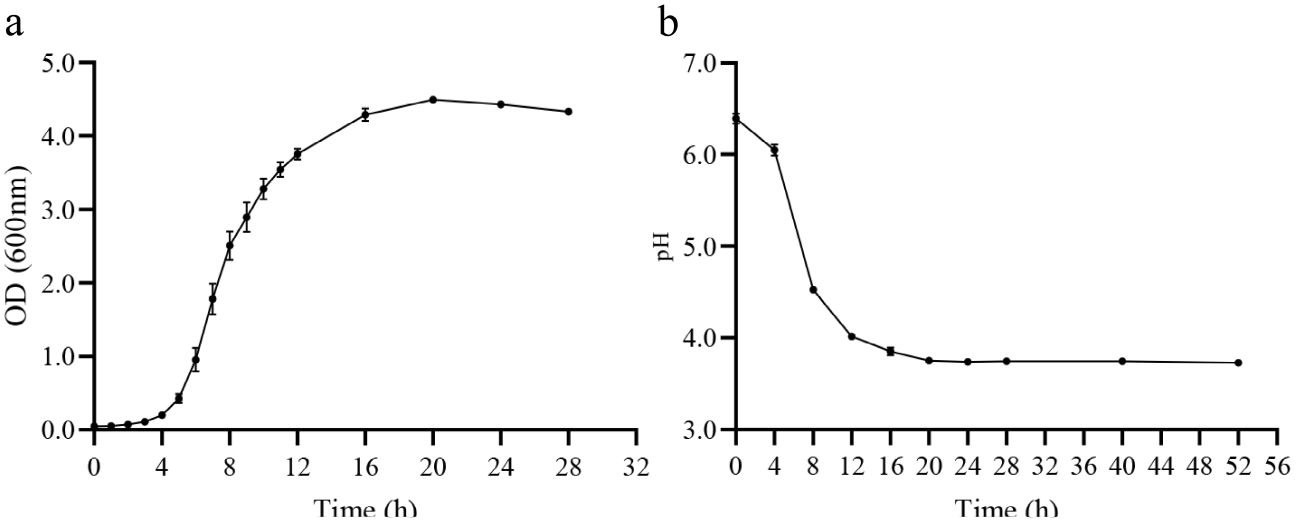

Table S1. The predicted prophage regions of *Lactiplantibacillus plantarum* strain JS21

Region	Length	Completeness	Score	Total Proteins	Region Position	Most Common Phage (Number of matching proteins)	GC %
1	18.1Kb	**intact**	100	25	36132-54235	PHAGE_Staphy_phiPV83_NC_002486(3)	42.15%
2	46Kb	**intact**	140	54	1154203-1200222	PHAGE_Lactob_Sha1_NC_019489(26)	40.70%
3	55.4Kb	**intact**	150	59	1812273-1867677	PHAGE_Lactob_Sha1_NC_019489(26)	40.65%
4	31.6Kb	**incomplete**	60	10	2137180-2168851	PHAGE_Paenib_Tripp_NC_028930(3)	43.31%
5	9.1Kb	**incomplete**	20	10	2186014-2195185	PHAGE_Strept_APCM01_NC_029030(1)	41.40%

Table S2. The first prophage (intact) region elements of *Lactiplantibacillus plantarum* JS21 (PHAGE_Staphy_phiPV83_NC_002486).

#	Locus	ORF Start	ORF Stop	Strand	Homolog/Ortholog Species	Homolog/Ortholog Protein	E-Value
1	PP_00028	36132	36581	Backward	Hypothetical protein	PP_00028, hypothetical protein, phage(gi100030), PHAGE_Lactob_PLE3_NC_031125	1.31E-17
2	PP_00029	36779	36979	Forward	Portal protein	PP_00029, portal protein, phage(gi100069), PHAGE_Vibrio_1.026.O._10N.222.49.C7_NC_049430	1.99E-08
3	PP_00030	37071	37295	Forward	Hypothetical protein	PP_00030, hypothetical	N/A
4	PP_00031	37513	38511	Forward	Transposase	PP_00031, putative transposase, phage(gi588498272), PHAGE_Staphy_StauST398_4_NC_023499	5.67E-63
5	*attL*	38584	38602	Forward	Attachment site(TATGATGGGCAGTCAGGGG)	*attL*	N/A
**6**	**PP_00032**	**38879**	**40033**	**Backward**	**Integrase**	**PP_00032, integrase, phage(gi23097608),PROPHAGE_Oceano_HTE831**	**3.25E-86**
7	PP_00033	40086	40652	Backward	Phage-like protein	PP_00033, putative transcriptional regulator, phage(gi446730276), PHAGE_Lactob_phiAQ113_NC_019782	4.25E-10
8	PP_00034	40969	41157	Forward	Phage-like protein	PP_00034, prophage Lp3 protein 4-like protein, phage(gi418489838), PHAGE_Lactob_Sha1_NC_019489	3.61E-08
9	PP_00035	41440	41658	Forward	Hypothetical protein	PP_00035, hypothetical	N/A
10	PP_00036	41655	42455	Forward	Hypothetical protein	PP_00036, hypothetical protein, phage(gi13095885), PHAGE_Lactoc_bIL310_NC_002669	7.03E-23
11	PP_00037	42455	43849	Forward	Tail protein	PP_00037, minor tail protein, phage(gi100055), PHAGE_Staphy_vB_SpsS_QT1_NC_048192	5.84E-77
12	PP_00038	43995	44474	Forward	Hypothetical protein	PP_00038, hypothetical	N/A
13	PP_00039	44489	44800	Forward	Hypothetical protein	PP_00039, hypothetical	N/A
14	PP_00040	44787	45128	Forward	Head protein	PP_00040, phage head-tail adaptor, phage(gi418489808), PHAGE_Lactob_Sha1_NC_019489	1.47E-14
15	PP_00041	45238	45510	Forward	Phage-like protein	PP_00041, phage endonuclease, phage(gi119443688), PHAGE_Staphy_Pvl108_NC_008689	1.19E-14
16	PP_00042	46414	46887	Forward	Phage-like protein	PP_00042, methyltransferase type 11, phage(gi100001), PHAGE_Gordon_Zirinka_NC_031097	2.34E-15
17	PP_00043	46884	48587	Forward	Hypothetical protein	PP_00043, hypothetical protein, phage(gi28876230), PHAGE_Strept_315.2_NC_004585	1.84E-132
18	PP_00044	48541	48741	Forward	Hypothetical protein	PP_00044, hypothetical	N/A
19	PP_00045	48742	49842	Forward	Portal protein	PP_00045, portal protein, phage(gi225626395), PHAGE_Entero_EFAP_1_NC_012419	4.20E-49
20	PP_00046	49839	51374	Forward	Head protein	PP_00046, capsid protein, phage(gi9635171), PHAGE_Staphy_PVL_NC_002321	1.00E-44
21	PP_00047	51488	51757	Forward	Head protein	PP_00047, head-tail joining protein, phage(gi225626391), PHAGE_Entero_EFAP_1_NC_012419	3.38E-08
22	PP_00048	51916	52284	Forward	Hypothetical protein	PP_00048, hypothetical	N/A
23	PP_00049	52408	52608	Forward	Portal protein	PP_00049, portal protein, phage(gi100069), PHAGE_Vibrio_1.026.O._10N.222.49.C7_NC_049430	7.45E-10
24	PP_00050	52695	52925	Forward	Hypothetical protein	PP_00050, hypothetical	N/A
25	PP_00051	52931	53044	Forward	Hypothetical protein	PP_00051, hypothetical	N/A
26	*attR*	53127	53145	Forward	Attachment site(TATGATGGGCAGTCAGGGG)	*attR*	N/A
27	PP_00052	53528	54235	Forward	Hypothetical protein	PP_00052, hypothetical protein, phage(gi100030), PHAGE_Altero_vB_AmeM_PT11_V22_NC_048847	3.00E-12

Table S3. The second prophage (intact) region elements of *Lactiplantibacillus plantarum* JS21 (PHAGE_Lactob_Sha1_NC_019489).

#	Locus	ORF Start	ORF Stop	Strand	Homolog/Ortholog Species	Homolog/Ortholog Protein	E-Value
1	PP_01075	1154203	1156470	Forward	Phage-like protein	PP_01075, ABC transporter, phage(gi371496158), PHAGE_Plankt_PaV_LD_NC_016564	4.26E-05
2	*attL*	1156982	1156998	Forward	Attachment site(AATTATGCCCCAGGCAG)	*attL*	N/A
**3**	**PP_01076**	**1157115**	**1158251**	**Backward**	**Integrase**	**PP_01076, phage integrase, phage(gi418489824), PHAGE_Lactob_Sha1_NC_019489**	**0**
4	PP_01077	1158655	1159170	Forward	Hypothetical protein	PP_01077, hypothetical protein, phage(gi100029), PHAGE_Pseudo_MD8_NC_031091	8.82E-07
5	PP_01078	1159471	1159647	Backward	Hypothetical protein	PP_01078, hypothetical	N/A
6	PP_01079	1159959	1161233	Backward	Hypothetical protein	PP_01079, hypothetical protein, phage(gi100036), PHAGE_Clostr_phiCDHM14_NC_048665	3.63E-28
7	PP_01080	1161290	1161697	Backward	Hypothetical protein	PP_01080, hypothetical protein, phage(gi100028), PHAGE_Lactob_PLE2_NC_031036	8.83E-39
8	PP_01081	1161690	1162022	Backward	Hypothetical protein	PP_01081, hypothetical protein, phage(gi100029), PHAGE_Lactob_PLE2_NC_031036	2.85E-37
9	PP_01082	1162280	1162501	Forward	Hypothetical protein	PP_01082, hypothetical	N/A
10	PP_01083	1162503	1163291	Forward	Phage-like protein	PP_01083, phage-related antirepressor, phage(gi418489833), PHAGE_Lactob_Sha1_NC_019489	1.07E-74
11	PP_01084	1163305	1163493	Forward	Phage-like protein	PP_01084, putative transcriptional regulator, phage(gi100045), PHAGE_Lactob_Lb_NC_047983	4.27E-06
12	PP_01085	1163490	1163891	Backward	Hypothetical protein	PP_01085, hypothetical	N/A
13	PP_01086	1164108	1164437	Forward	Hypothetical protein	PP_01086, hypothetical protein, phage(gi418489836), PHAGE_Lactob_Sha1_NC_019489	5.81E-72
14	PP_01087	1164527	1164781	Forward	Hypothetical protein	PP_01087, hypothetical	N/A
15	PP_01088	1164951	1165121	Forward	Hypothetical protein	PP_01088, hypothetical	N/A
16	PP_01089	1165121	1165981	Forward	Hypothetical protein	PP_01089, hypothetical protein, phage(gi971754947), PHAGE_Lactob_iLp1308_NC_028911	1.59E-48
17	PP_01090	1165982	1166644	Forward	Phage-like protein	PP_01090, phage nucleotide-binding protein, phage(gi418489844), PHAGE_Lactob_Sha1_NC_019489	9.83E-162
18	PP_01091	1166646	1167305	Forward	Hypothetical protein	PP_01091, hypothetical protein, phage(gi418489845), PHAGE_Lactob_Sha1_NC_019489	1.58E-156
19	PP_01092	1167352	1168044	Forward	Hypothetical protein	PP_01092, hypothetical protein, phage(gi418489846), PHAGE_Lactob_Sha1_NC_019489	2.96E-172
20	PP_01093	1168150	1168266	Forward	Hypothetical protein	PP_01093, hypothetical	N/A
21	PP_01094	1168273	1169022	Backward	Hypothetical protein	PP_01094, hypothetical protein, phage(gi418489416), PHAGE_Lactob_LF1_NC_019486	4.38E-42
22	PP_01095	1169090	1169896	Forward	Hypothetical protein	PP_01095, hypothetical protein, phage(gi238821328), PHAGE_Strept_PH10_NC_012756	9.65E-38
23	PP_01096	1169890	1170825	Forward	Phage-like protein	PP_01096, DNA replication protein, phage(gi971747669), PHAGE_Lactob_iA2_NC_028830	6.68E-55
24	PP_01097	1171121	1171429	Forward	Hypothetical protein	PP_01097, hypothetical protein, phage(gi418489790), PHAGE_Lactob_Sha1_NC_019489	1.10E-61
25	PP_01098	1171422	1171538	Forward	Hypothetical protein	PP_01098, hypothetical protein, phage(gi418489791), PHAGE_Lactob_Sha1_NC_019489	9.77E-09
26	PP_01099	1171773	1171886	Forward	Phage-like protein	PP_01099, LP1-like protein, phage(gi418489797), PHAGE_Lactob_Sha1_NC_019489	3.42E-16
27	PP_01100	1171867	1172292	Forward	Phage-like protein	PP_01100, phage transcriptional activator RinA, phage(gi418489798), PHAGE_Lactob_Sha1_NC_019489	1.10E-77
28	PP_01101	1172733	1172912	Forward	Hypothetical protein	PP_01101, hypothetical	N/A

29	PP_01102	1172917	1173870	Backward	Hypothetical protein	PP_01102, hypothetical	N/A
30	PP_01103	1174484	1174996	Forward	Hypothetical protein	PP_01103, hypothetical	N/A
31	PP_01104	1175491	1175949	Forward	Terminase	PP_01104, terminase small subunit, phage(gi422934327), PHAGE_Bacill_phIS3501_NC_019502	3.34E-22
32	PP_01105	1175936	1177726	Forward	Terminase	PP_01105, putative terminase large subunit, phage(gi418489114), PHAGE_Lactob_JCL1032_NC_019456	0
33	PP_01106	1177746	1178975	Forward	Portal protein	PP_01106, portal protein, phage(gi985759335), PHAGE_Bacter_Rani_NC_029084	1.85E-141
34	PP_01107	1178947	1179666	Forward	Protease	PP_01107, ATP-dependent Clp protease proteolytic subunit, phage(gi971740652), PHAGE_Paenib_HB10c2_NC_028758	1.16E-86
35	PP_01108	1179669	1180814	Forward	Head protein	PP_01108, major capsid protein, phage(gi971482315), PHAGE_Paenib_Harrison_NC_028746	5.95E-115
36	PP_01109	1180833	1180994	Forward	Hypothetical protein	PP_01109, hypothetical	N/A
37	PP_01110	1181047	1181391	Forward	Phage-like protein	PP_01110, phage protein DNA packaging protein, phage(gi418489807), PHAGE_Lactob_Sha1_NC_019489	2.59E-07
38	PP_01111	1181375	1181737	Forward	Head protein	PP_01111, phage head-tail adaptor, phage(gi418489808), PHAGE_Lactob_Sha1_NC_019489	1.30E-83
39	PP_01112	1181727	1182167	Forward	Head protein	PP_01112, phage head-tail joining protein, phage(gi418489809), PHAGE_Lactob_Sha1_NC_019489	1.04E-99
40	PP_01113	1182164	1182547	Forward	Hypothetical protein	PP_01113, hypothetical protein, phage(gi418489810), PHAGE_Lactob_Sha1_NC_019489	6.70E-84
41	PP_01114	1182548	1183186	Forward	Tail protein	PP_01114, major tail protein, phage(gi418489811), PHAGE_Lactob_Sha1_NC_019489	1.13E-144
42	PP_01116	1183213	1183329	Backward	Hypothetical protein	PP_01116, hypothetical	N/A
43	PP_01115	1183388	1183771	Forward	Hypothetical protein	PP_01115, hypothetical protein, phage(gi418489812), PHAGE_Lactob_Sha1_NC_019489	3.88E-85
44	PP_01117	1183768	1183959	Forward	Hypothetical protein	PP_01117, hypothetical protein, phage(gi418489813), PHAGE_Lactob_Sha1_NC_019489	3.71E-39
45	PP_01118	1183972	1189194	Forward	Tail protein	PP_01118, minor tail protein, phage(gi418489814), PHAGE_Lactob_Sha1_NC_019489	0
46	PP_01119	1189267	1191042	Forward	Phage-like protein	PP_01119, minor structural protein gp75-like protein, phage(gi418489815), PHAGE_Lactob_Sha1_NC_019489	0
47	PP_01120	1191108	1193522	Forward	Phage-like protein	PP_01120, minor structural protein gp89-like protein, phage(gi418489816), PHAGE_Lactob_Sha1_NC_019489	0
48	PP_01121	1193539	1195824	Forward	Hypothetical protein	PP_01121, hypothetical protein, phage(gi100085), PHAGE_Lactob_Satyr_NC_047918	0
49	PP_01122	1195802	1196053	Forward	Hypothetical protein	PP_01122, hypothetical protein, phage(gi418489818), PHAGE_Lactob_Sha1_NC_019489	1.68E-35
50	PP_01123	1196057	1196218	Forward	Hypothetical protein	PP_01123, hypothetical protein, phage(gi418489819), PHAGE_Lactob_Sha1_NC_019489	1.89E-25
51	PP_01124	1196202	1197299	Forward	Phage-like protein	PP_01124, prophage Lp2 protein 53-like protein, phage(gi418489820), PHAGE_Lactob_Sha1_NC_019489	3.98E-86
52	PP_01125	1197296	1197511	Forward	Hypothetical protein	PP_01125, hypothetical protein, phage(gi985757750), PHAGE_Lactob_LfeSau_NC_029068	6.53E-28
53	PP_01126	1197525	1198697	Forward	Phage-like protein	PP_01126, endolysin, phage(gi418489821), PHAGE_Lactob_Sha1_NC_019489	0
54	PP_01127	1198697	1198960	Forward	Phage-like protein	PP_01127, phage-related holin, phage(gi418489822), PHAGE_Lactob_Sha1_NC_019489	2.10E-55
55	PP_01128	1198973	1199503	Forward	Hypothetical protein	PP_01128, hypothetical protein, phage(gi418489823), PHAGE_Lactob_Sha1_NC_019489	1.22E-77
56	*attR*	1200222	1200238	Forward	Attachment site(AATTATGCCCCAGGCAG)	*attR*	N/A

Table S4. The third prophage (intact) region elements of *Lactiplantibacillus plantarum* JS21 (PHAGE_Lactob_Sha1_NC_019489).

#	Locus	ORF Start	ORF Stop	Strand	Homolog/Ortholog Species	Homolog/Ortholog Protein	E-Value
1	*attL*	1812273	1812284	Forward	Attachment site(AAACCAAAAAGC)	*attL*	N/A
2	PP_01770	1824504	1824878	Backward	Hypothetical protein	PP_01770, hypothetical protein, phage(gi100097), PHAGE_Lactob_Lenus_NC_047897	8.54E-46
3	PP_01771	1824891	1825154	Backward	Phage-like protein	PP_01771, phage-related holin, phage(gi418489822), PHAGE_Lactob_Sha1_NC_019489	7.74E-54
4	PP_01772	1825154	1826179	Backward	Phage-like protein	PP_01772, endolysin, phage(gi418489821), PHAGE_Lactob_Sha1_NC_019489	0
5	PP_01773	1826191	1826436	Backward	Hypothetical protein	PP_01773, hypothetical protein, phage(gi985757750), PHAGE_Lactob_LfeSau_NC_029068	1.89E-28
6	PP_01774	1826433	1827797	Backward	Phage-like protein	PP_01774, prophage Lp2 protein 53-like protein, phage(gi418489820), PHAGE_Lactob_Sha1_NC_019489	7.00E-78
7	PP_01775	1827781	1827942	Backward	Hypothetical protein	PP_01775, hypothetical protein, phage(gi418489819), PHAGE_Lactob_Sha1_NC_019489	3.05E-28
8	PP_01776	1827946	1828188	Backward	Hypothetical protein	PP_01776, hypothetical protein, phage(gi418489818), PHAGE_Lactob_Sha1_NC_019489	6.94E-41
9	PP_01777	1828181	1830976	Backward	Hypothetical protein	PP_01777, hypothetical protein, phage(gi100085), PHAGE_Lactob_Satyr_NC_047918	0
10	PP_01778	1830993	1833407	Backward	Phage-like protein	PP_01778, minor structural protein gp89-like protein, phage(gi418489816), PHAGE_Lactob_Sha1_NC_019489	0
11	PP_01779	1833473	1835245	Backward	Phage-like protein	PP_01779, minor structural protein gp75-like protein, phage(gi418489815), PHAGE_Lactob_Sha1_NC_019489	0
12	PP_01780	1835305	1840203	Backward	Tail protein	PP_01780, putative tail component protein, phage(gi29165636), PHAGE_Strept_DT1_NC_002072	0
13	PP_01781	1840235	1840426	Backward	Hypothetical protein	PP_01781, hypothetical protein, phage(gi418489813), PHAGE_Lactob_Sha1_NC_019489	3.26E-07
14	PP_01782	1840465	1840839	Backward	Hypothetical protein	PP_01782, hypothetical	N/A
15	PP_01783	1840914	1841567	Backward	Tail protein	PP_01783, putative major tail protein, phage(gi48697269), PHAGE_Lactob_phiAT3_NC_005893	2.59E-22
16	PP_01784	1841583	1841963	Backward	Tail protein	PP_01784, putative tail component, phage(gi48697268), PHAGE_Lactob_phiAT3_NC_005893	2.60E-16
17	PP_01785	1841963	1842370	Backward	Tail protein	PP_01785, putative tail component protein, phage(gi9632427), PHAGE_Strept_DT1_NC_002072	2.08E-35
18	PP_01786	1842373	1842720	Backward	Head protein	PP_01786, putative head-tail joining protein, phage(gi9632902), PHAGE_Strept_Sfi19_NC_000871	2.74E-16
19	PP_01787	1842710	1843042	Backward	Phage-like protein	PP_01787, phage protein DNA packaging protein, phage(gi418489807), PHAGE_Lactob_Sha1_NC_019489	4.77E-55
20	PP_01788	1843114	1844346	Backward	Head protein	PP_01788, HK97 family phage major capsid protein, phage(gi418489806), PHAGE_Lactob_Sha1_NC_019489	0
21	PP_01789	1844346	1845110	Backward	Protease	PP_01789, protease subunit of ATP-dependent Clp protease, phage(gi418489805), PHAGE_Lactob_Sha1_NC_019489	1.37E-170
22	PP_01790	1845088	1846251	Backward	Portal protein	PP_01790, phage portal protein, phage(gi418489804), PHAGE_Lactob_Sha1_NC_019489	0
23	PP_01791	1846254	1846448	Backward	Head protein	PP_01791, phage head-tail joining protein, phage(gi418489803), PHAGE_Lactob_Sha1_NC_019489	6.73E-35
24	PP_01792	1846438	1847805	Backward	Terminase	PP_01792, phage terminase-like protein large subunit, phage(gi418489802),	0

						PHAGE_Lactob_Sha1_NC_019489	
25	PP_01793	1847926	1849101	Forward	Transposase	PP_01793, transposase, phage(gi26246249), PROPHAGE_Escher_CFT073	2.25E-62
26	PP_01794	1849133	1849696	Backward	Terminase	PP_01794, phage terminase-like protein large subunit, phage(gi418489802), PHAGE_Lactob_Sha1_NC_019489	5.27E-104
27	PP_01795	1849706	1850161	Backward	Terminase	PP_01795, P27 family phage terminase small subunit, phage(gi418489801), PHAGE_Lactob_Sha1_NC_019489	2.19E-106
28	PP_01796	1850351	1850602	Backward	Hypothetical protein	PP_01796, hypothetical	N/A
29	PP_01797	1850620	1851036	Backward	Hypothetical protein	PP_01797, hypothetical	N/A
30	PP_01798	1851042	1851512	Backward	Phage-like protein	PP_01798, restriction endonuclease, phage(gi418489800), PHAGE_Lactob_Sha1_NC_019489	7.09E-103
31	PP_01799	1851523	1851693	Backward	Phage-like protein	PP_01799, HNH nuclease, phage(gi418489799), PHAGE_Lactob_Sha1_NC_019489	1.66E-32
32	PP_01800	1851865	1852803	Backward	Hypothetical protein	PP_01800, hypothetical	N/A
33	PP_01801	1853073	1853498	Backward	Phage-like protein	PP_01801, phage transcriptional activator RinA, phage(gi418489798), PHAGE_Lactob_Sha1_NC_019489	2.44E-86
34	PP_01802	1853479	1853649	Backward	Phage-like protein	PP_01802, LP1-like protein, phage(gi418489797), PHAGE_Lactob_Sha1_NC_019489	1.86E-27
35	PP_01803	1853642	1854118	Backward	Hypothetical protein	PP_01803, hypothetical protein, phage(gi100142), PHAGE_Lactob_Iacchus_NC_048084	2.20E-63
36	PP_01804	1854115	1854531	Backward	Head protein	PP_01804, capsid maturation protease, phage(gi100067), PHAGE_Lactob_Lpa804_NC_048134	2.80E-41
37	PP_01805	1854555	1854704	Backward	Hypothetical protein	PP_01805, hypothetical	N/A
38	PP_01806	1854750	1854863	Backward	Hypothetical protein	PP_01806, hypothetical protein, phage(gi418489791), PHAGE_Lactob_Sha1_NC_019489	3.19E-07
39	PP_01807	1854856	1855236	Backward	Phage-like protein	PP_01807, gp63, phage(gi16798850), PHAGE_Lister_A118_NC_003216	1.53E-35
40	PP_01808	1855233	1855733	Backward	Hypothetical protein	PP_01808, hypothetical protein, phage(gi23455787), PHAGE_Lactob_phig1e_NC_004305	1.19E-75
41	PP_01809	1855869	1856654	Backward	Phage-like protein	PP_01809, DNA replication protein, phage(gi418489789), PHAGE_Lactob_Sha1_NC_019489	2.69E-175
42	PP_01810	1856654	1857421	Backward	Fiber protein	PP_01810, tail fiber protein, phage(gi100052), PHAGE_Bacill_vB_BhaS_171_NC_030904	1.58E-50
43	PP_01811	1858047	1858220	Backward	Hypothetical protein	PP_01811, hypothetical protein, phage(gi418489841), PHAGE_Lactob_Sha1_NC_019489	5.78E-30
44	PP_01812	1858232	1858432	Backward	Hypothetical protein	PP_01812, hypothetical protein, phage(gi418489840), PHAGE_Lactob_Sha1_NC_019489	5.20E-36
45	PP_01813	1858435	1858683	Backward	Hypothetical protein	PP_01813, hypothetical protein, phage(gi418489839), PHAGE_Lactob_Sha1_NC_019489	8.76E-41
46	PP_01814	1859949	1860209	Backward	Hypothetical protein	PP_01814, hypothetical	N/A
47	PP_01815	1860267	1860488	Forward	Hypothetical protein	PP_01815, hypothetical	N/A
48	PP_01816	1860483	1860731	Backward	Hypothetical protein	PP_01816, hypothetical	N/A
49	PP_01817	1860745	1860954	Backward	Hypothetical protein	PP_01817, hypothetical protein, phage(gi9633008), PHAGE_Lactob_phiadh_NC_000896	9.80E-06
50	PP_01818	1860966	1861673	Backward	Phage-like protein	PP_01818, phage-related antirepressor, phage(gi418489833), PHAGE_Lactob_Sha1_NC_019489	1.43E-36
51	PP_01819	1861730	1861975	Forward	Hypothetical protein	PP_01819, hypothetical	N/A

52	PP_01820	1862264	1862479	Backward	Hypothetical protein	PP_01820, hypothetical	N/A
53	PP_01821	1862736	1863068	Forward	Hypothetical protein	PP_01821, hypothetical protein, phage(gi100029), PHAGE_Lactob_PLE2_NC_031036	7.11E-40
54	PP_01822	1863061	1863468	Forward	Hypothetical protein	PP_01822, hypothetical protein, phage(gi100028), PHAGE_Lactob_PLE2_NC_031036	6.39E-39
55	PP_01823	1863529	1864305	Forward	Hypothetical protein	PP_01823, hypothetical	N/A
56	PP_01824	1864317	1864517	Forward	Hypothetical protein	PP_01824, hypothetical protein, phage(gi418489829), PHAGE_Lactob_Sha1_NC_019489	4.61E-36
57	PP_01825	1864827	1865009	Forward	Hypothetical protein	PP_01825, hypothetical	N/A
58	PP_01826	1865199	1865513	Forward	Hypothetical protein	PP_01826, hypothetical	N/A
59	PP_01827	1865710	1866285	Backward	Hypothetical protein	PP_01827, hypothetical	N/A
60	*attR*	1866346	1866357	Forward	Attachment site(AAACCAAAAAGC)	*attR*	N/A
**61**	**PP_01828**	**1866514**	**1867677**	**Forward**	**Integrase**	**PP_01828, phage integrase, phage(gi418489411), PHAGE_Lactob_LF1_NC_019486**	**5.03E-79**

Table S5. The fourth prophage (incomplete) region elements of *Lactiplantibacillus plantarum* JS21 (PHAGE_Paenib_Tripp_NC_028930).

#	Locus	ORF Start	ORF Stop	Strand	Homolog/Ortholog Species	Homolog/Ortholog Protein	E-Value
1	*attL*	2137180	2137192	Forward	Attachment site(TTTGGAAAACAAA)	*attL*	N/A
2	PP_02105	2151081	2152970	Backward	Phage-like protein	PP_02105, ABC transporter, phage(gi9630145), PHAGE_Bacill_SPbeta_NC_001884	1.07E-30
3	PP_02106	2152970	2154700	Backward	Phage-like protein	PP_02106, ABC transporter, phage(gi9630145), PHAGE_Bacill_SPbeta_NC_001884	4.72E-18
4	PP_02107	2154954	2155814	Backward	Plate protein	PP_02107, putative baseplate hub protein, phage(gi100137), PHAGE_Escher_RCS47_NC_042128	3.77E-05
5	PP_02108	2155841	2156293	Backward	Hypothetical protein	PP_02108, hypothetical protein, phage(gi971746471), PHAGE_Staphy_StB20_like_NC_028821	9.01E-06
6	PP_02109	2156409	2156840	Backward	Transposase	PP_02109, transposase, phage(gi971756981), PHAGE_Paenib_Tripp_NC_028930	4.05E-27
7	PP_02110	2156813	2157184	Backward	Transposase	PP_02110, transposase, phage(gi971756982), PHAGE_Paenib_Tripp_NC_028930	4.19E-36
8	PP_02111	2157189	2157971	Forward	Phage-like protein	PP_02111, chromosome partitioning ATPase, phage(gi418489414), PHAGE_Lactob_LF1_NC_019486	8.72E-19
9	PP_02112	2158186	2158353	Forward	Hypothetical protein	PP_02112, hypothetical	N/A
10	PP_02113	2158353	2159060	Forward	Hypothetical protein	PP_02113, hypothetical	N/A
**11**	**PP_02114**	**2159223**	**2160344**	**Forward**	**Integrase**	**PP_02114, putative integrase, phage(gi937456703), PHAGE_Lactob_LBR48_NC_027990**	**1.12E-52**
12	*attR*	2168851	2168863	Forward	Attachment site(TTTGGAAAACAAA)	*attR*	N/A

Table S6. The fifth prophage (incomplete) region elements of *Lactiplantibacillus plantarum* JS21 (PHAGE_Strept_APCM01_NC_029030).

#	Locus	ORF Start	ORF Stop	Strand	Homolog/Ortholog Species	Homolog/Ortholog Protein	E-Value
1	*attL*	2186014	2186033	Forward	Attachment site(CTCGCCATCTCCATTATTGA)	*attL*	N/A
2	PP_02143	2188064	2188438	Backward	Hypothetical protein	PP_02143, hypothetical protein, phage(gi966201497), PHAGE_Strept_T12_NC_028700	1.94E-13
3	PP_02144	2188725	2190140	Backward	Hypothetical protein	PP_02144, hypothetical protein, phage(gi100019), PHAGE_Clostr_phiCT453A_NC_028991	1.64E-82
4	PP_02145	2190152	2190931	Backward	Hypothetical protein	PP_02145, hypothetical protein, phage(gi966198623), PHAGE_Strept_Str_PAP_1_NC_028666	1.85E-27
5	PP_02146	2190944	2191165	Backward	Hypothetical protein	PP_02146, hypothetical	N/A
6	PP_02147	2191158	2191580	Backward	Hypothetical protein	PP_02147, hypothetical	N/A
7	PP_02148	2191722	2191835	Backward	Hypothetical protein	PP_02148, hypothetical	N/A
8	PP_02149	2191893	2192159	Backward	Hypothetical protein	PP_02149, hypothetical	N/A
9	PP_02150	2192327	2193013	Backward	Phage-like protein	PP_02150, Orf3, phage(gi13095661), PHAGE_Lactoc_bIL311_NC_002670	4.99E-19
10	PP_02151	2193341	2193835	Forward	Phage-like protein	PP_02151, putative transcriptional regulator, phage(gi446730276), PHAGE_Lactob_phiAQ113_NC_019782	2.17E-19
**11**	**PP_02152**	**2193885**	**2195051**	**Forward**	**Integrase**	**PP_02152, integrase, phage(gi23097608), PROPHAGE_Oceano_HTE831**	**2.64E-95**
12	*attR*	2195185	2195204	Forward	Attachment site(CTCGCCATCTCCATTATTGA)	*attR*	N/A

Table S7. Horizontal gene transfer of prophage region proteins using ProteinBLAST

S/N	#	Locus	ORF Start	ORF Stop	Strand	Homolog/Ortholog Species	Protein BLAST	Accession	E-value
The first prophage (intact) region elements of *Lactiplantibacillus plantarum* strain JS21									
1	1	PP_00028	36132	36581	Backward	Hypothetical protein	pyridoxamine 5'-phosphate oxidase family protein [*Lactiplantibacillus argentoratensis*]	WP_214334341.1	7E-103
2	4	PP_00031	37513	38511	Forward	Transposase	IS30 family transposase [*Lactiplantibacillus argentoratensis*]	MBT1142305.1	0.0
3	6	PP_00032	38879	40033	Backward	Integrase	site-specific integrase [*Lactiplantibacillus nangangensis*]	WP_137615032.1	0.0
4	7	PP_00033	40086	40652	Backward	Phage-like protein	helix-turn-helix domain-containing protein [*Lentilactobacillus buchneri*]	WP_172884887.1	5E-30
5	11	PP_00037	42455	43849	Forward	Tail protein	virulence-associated E family protein [*Levilactobacillus brevis*]	WP_087609387.1	0.0
6	19	PP_00045	48742	49842	Forward	Portal protein	phage portal protein [*Lactiplantibacillus nangangensis*]	WP_137615043.1	0.0
7	20	PP_00046	49839	51374	Forward	Head protein	phage major capsid protein [*Lactiplantibacillus nangangensis*]	WP_137615044.1	0.0
8	21	PP_00047	51488	51757	Forward	Head protein	head-tail connector protein [*Lactiplantibacillus xiangfangensis*]	WP_057705766.1	1e-56
9	22	PP_00048	51916	52284	Forward	Hypothetical protein	hypothetical protein [*Limosilactobacillus reuteri*]	WP_264508898.1	2e-78
10	23	PP_00049	52408	52608	Forward	Portal protein	cold-shock protein [*Limosilactobacillus reuteri*]	WP_169477496.1	3e-37
11	25	PP_00051	52931	53044	Forward	Hypothetical protein	TPA: hypothetical protein [*Caudoviricetes* sp.]	DAZ29239.1	5E-14
The second prophage (intact) region elements of *Lactiplantibacillus plantarum* strain JS21									
12	1	PP_01075	1154203	1156470	Forward	Phage-like protein	excinuclease ABC subunit UvrA [*Lactiplantibacillus argentoratensis*]	WP_225588668.1	0.00E+00
13	4	PP_01077	1158655	1159170	Forward	Hypothetical protein	DUF2335 domain-containing protein [*Levilactobacillus brevis*]	WP_015474456.1	1.00E-101
14	5	PP_01078	1159471	1159647	Backward	Hypothetical protein	exonuclease domain-containing protein [*Levilactobacillus fuyuanensis*]	WP_125700023.1	5.00E-120
15	11	PP_01084	1163305	1163493	Forward	Phage-like protein	hypothetical protein [*Lactiplantibacillus argentoratensis*]	WP_285209501.1	3.00E-09
16	13	PP_01086	1164108	1164437	Forward	Hypothetical protein	DUF771 domain-containing protein [*Lactiplantibacillus argentoratensis*]	WP_214334453.1	1.00E-73
17	14	PP_01087	1164527	1164781	Forward	Hypothetical protein	hypothetical protein LJA01_21400 [*Lactobacillus japonicus*]	GEK64237.1	3.00E-48
18	18	PP_01091	1166646	1167305	Forward	Hypothetical protein	DUF669 domain-containing protein [*Lactiplantibacillus argentoratensis*]	WP_260354589.1	7.00E-155
19	19	PP_01092	1167352	1168044	Forward	Hypothetical protein	hypothetical protein LJA01_21460 [*Lactobacillus japonicus*]	GEK64243.1	4.00E-171
20	21	PP_01094	1168273	1169022	Backward	Hypothetical protein	DUF4393 domain-containing protein [*Lactobacillaceae*]	WP_187357782.1	2.00E-54
21	22	PP_01095	1169090	1169896	Forward	Hypothetical protein	phage replisome organizer N-terminal domain-containing protein [*Lactiplantibacillus argentoratensis*]	WP_214417724.1	0.00E+00
22	23	PP_01096	1169890	1170825	Forward	Phage-like protein	ATP-binding protein [*Lactiplantibacillus argentoratensis*]	WP_244975215.1	0.00E+00
23	24	PP_01097	1171121	1171429	Forward	Hypothetical protein	hypothetical protein [*Lactiplantibacillus fabifermentans*]	WP_033614079.1	2.00E-63
24	25	PP_01098	1171422	1171538	Forward	Hypothetical protein	hypothetical protein LJA01_21510 [*Lactobacillus japonicus*]	GEK64248.1	3.00E-14
25	26	PP_01099	1171773	1171886	Forward	Phage-like protein	hypothetical protein [*Lactiplantibacillus mudanjiangensis*]	WP_130846690.1	1.00E-14

26	27	PP_01100	1171867	1172292	Forward	Phage-like protein	transcriptional regulator [*Lactiplantibacillus argentoratensis*]	WP_253290706.1	4.00E-94
27	28	PP_01101	1172733	1172912	Forward	Hypothetical protein	hypothetical protein [*Weissella confusa*]	WP_199402017.1	2.00E-08
28	29	PP_01102	1172917	1173870	Backward	Hypothetical protein	hypothetical protein [*Weissella cibaria*]	WP_261721978.1	1.00E-95
29	31	PP_01104	1175491	1175949	Forward	Terminase	phage terminase small subunit P27 family [*Lactiplantibacillus mudanjiangensis*]	WP_130852332.1	1.00E-101
30	32	PP_01105	1175936	1177726	Forward	Terminase	terminase large subunit [*Levilactobacillus brevis*]	WP_069359847.1	0
31	33	PP_01106	1177746	1178975	Forward	Portal protein	phage portal protein [*Levilactobacillus brevis*]	WP_024526686.1	0.00E+00
32	34	PP_01107	1178947	1179666	Forward	Protease	Clp protease ClpP [*Levilactobacillus brevis*]	WP_060416649.1	8.00E-170
33	35	PP_01108	1179669	1180814	Forward	Head protein	phage major capsid protein [*Levilactobacillus brevis*]	WP_060416648.1	0.00E+00
34	37	PP_01110	1181047	1181391	Forward	Phage-like protein	head-tail connector protein [*Levilactobacillus brevis*]	WP_024526689.1	3.00E-70
35	40	PP_01113	1182164	1182547	Forward	Hypothetical protein	tail protein [*Lactobacillus* phage Sha1]	YP_007003562.1	4.00E-82
36	42	PP_01116	1183213	1183329	Backward	Hypothetical protein	TPA: hypothetical protein [*Siphoviridae* sp. ctk5O4]	DAF51228.1	0.019
37	43	PP_01115	1183388	1183771	Forward	Hypothetical protein	hypothetical protein F2048_23865 [*Bacteroides fragilis*]	KAA4797547.1	9.00E-83
38	44	PP_01117	1183768	1183959	Forward	Hypothetical protein	hypothetical protein [*Loigolactobacillus backii*]	WP_143452801.1	9.00E-22
39	51	PP_01124	1196202	1197299	Forward	Phage-like protein	collagen-like protein [*Pediococcus ethanolidurans*]	WP_216762492.1	0.00E+00
40	52	PP_01125	1197296	1197511	Forward	Hypothetical protein	hypothetical protein [*Levilactobacillus brevis*]	WP_216577569.1	6.00E-39
41	53	PP_01126	1197525	1198697	Forward	Phage-like protein	GH25 family lysozyme [*Lactiplantibacillus argentoratensis*]	WP_210696722.1	0.00E+00
42	55	PP_01128	1198973	1199503	Forward	Hypothetical protein	hypothetical protein [*Lactiplantibacillus fabifermentans*]	WP_056993534.1	1.00E-111
The third prophage (intact) region elements of *Lactiplantibacillus plantarum* strain JS21									
43	2	PP_01770	1824504	1824878)	Backward	Hypothetical protein	phage holin [*Lactiplantibacillus argentoratensis*]	WP_216491151.1	6.00E-74
44	5	PP_01773	1826191	1826436)	Backward	Hypothetical protein	hypothetical protein [*Lactiplantibacillus xiangfangensis*]	WP_057705566.1	1.00E-70
45	7	PP_01775	1827781	1827942)	Backward	Hypothetical protein	hypothetical protein [*Gluconobacter oxydans*]	WP_253441138.1	8.00E-48
46	9	PP_01777	1828181	1830976)	Backward	Hypothetical protein	phage tail protein [*Lactiplantibacillus argentoratensis*]	WP_216491148.1	0.00E+00
47	12	PP_01780	1835305	1840203)	Backward	Tail protein	tape measure protein [*Lactobacillus japonicus*]	GEK64269.1	0.00E+00
48	14	PP_01782	1840465	1840839)	Backward	Hypothetical protein	phage tail assembly chaperone [*Lactiplantibacillus mudanjiangensis*]	WP_130851912.1	9.00E-80
49	17	PP_01785	1841963	1842370)	Backward	Tail protein	hypothetical protein LJA01_21670 [*Lactobacillus japonicus*]	GEK64264.1	4.00E-87
50	19	PP_01787	1842710	1843042)	Backward	Phage-like protein	head-tail connector protein [*Lactiplantibacillus mudanjiangensis*]	WP_130851908.1	3.00E-64
51	23	PP_01791	1846254	1846448)	Backward	Head protein	DUF1056 family protein [*Lactiplantibacillus argentoratensis*]	WP_285209523.1	6.00E-34
52	24	PP_01792	1846438	1847805)	Backward	Terminase	terminase large subunit [*Pediococcus acidilactici*]	WP_159218408.1	0.00E+00
53	25	PP_01793	1847926	1849101	Forward	Transposase	IS256 family transposase [*Fructilactobacillus sanfranciscensis*]	WP_238979325.1	0.00E+00
54	26	PP_01794	1849133	1849696)	Backward	Terminase	terminase large subunit [*Bacteroides fragilis*]	KAA4797499.1	8.00E-123

55	29	PP_01797	1850620	1851036)	Backward	Hypothetical protein	hypothetical protein [*Levilactobacillus brevis*]	WP_060463300.1	1.00E-62
56	31	PP_01799	1851523	1851693)	Backward	Phage-like protein	hypothetical protein [*Lactiplantibacillus argentoratensis*]	WP_214334474.1	2e-30
57	33	PP_01801	1853073	1853498)	Backward	Phage-like protein	RinA family transcriptional regulator [*Lactiplantibacillus fabifermentans* DSM 21115]	KRO24919.1	3e-89
58	36	PP_01804	1854115	1854531)	Backward	Head protein	YopX family protein [*Lactiplantibacillus argentoratensis*]	MBU5277831.1	2e-82
59	40	PP_01808	1855233	1855733)	Backward	Hypothetical protein	hypothetical protein [*Lentilactobacillus parabuchneri*]	WP_301521624.1	7e-94
60	41	PP_01809	1855869	1856654)	Backward	Phage-like protein	ATP-binding protein [*Lactiplantibacillus argentoratensis*]	WP_216491080.1	0.0
61	46	PP_01814	1859949	1860209	Backward	Hypothetical protein	hypothetical protein DY78_GL000849 [*Lactiplantibacillus fabifermentans* DSM 21115]	KRO26535.1	2e-40
62	51	PP_01819	1861730	1861975	Forward	Hypothetical protein	hypothetical protein [*Limosilactobacillus reuteri*]	WP_143449772.1	8e-43
63	56	PP_01824	1864317	1864517	Forward	Hypothetical protein	capsule polysaccharide transpoter [*Lactobacillus delbrueckii* subsp. *bulgaricus*]	MBT9072018.1	4e-06
64	57	PP_01825	1864827	1865009	Forward	Hypothetical protein	hypothetical protein [*Lactiplantibacillus argentoratensis*]	WP_285209498.1	2e-32
The fourth prophage (incomplete) region elements of *Lactiplantibacillus plantarum* strain JS21									
65	2	PP_02105	2151081	2152970	Backward	Phage-like protein	Select seq dbj|GEK64508.1| multidrug ABC transporter ATP-binding protein [*Lactobacillus japonicus*]	GEK64508.1	0.0
66	4	PP_02107	2154954	2155814	Backward	Plate protein	IS982 family transposase [*Levilactobacillus parabrevis*]	WP_260340338.1	0.0
67	6	PP_02109	2156409	2156840	Backward	Transposase	Transposase [*Levilactobacillus brevis* ATCC 367]	ABJ64411.1	2e-102
68	7	PP_02110	2156813	2157184	Backward	Transposase	Transposase [*Levilactobacillus brevis* ATCC 367]	ABJ64410.1	5e-84
69	8	PP_02111	2157189	2157971	Forward	Phage-like protein	ParA family protein [*Latilactobacillus curvatus*]	WP_221276490.1	2e-108
70	10	PP_02113	2158353	2159060	Forward	Hypothetical protein	Select seq ref|WP_139960858.1| hypothetical protein [*Levilactobacillus brevis*]	WP_139960858.1	1e-67
The fifth prophage (incomplete) region elements of *Lactiplantibacillus plantarum* strain JS21									
71	8	PP_02149	2191893	2192159	Backward	Hypothetical protein	helix-turn-helix domain-containing protein [*Lactiplantibacillus mudanjiangensis*]	WP_130846642.1	2e-30

Table S8. The predicted transposases of the JS21 genome by using IS Finder

#	Sequences producing significant alignments	IS Family	Group	Origin	Score(bits)	E. value
1	ISP2	IS1182		*Lactobacillus plantarum*	3356	0
2	ISP1	ISL3		*Lactobacillus plantarum*	2809	0
3	IS1310	IS256		*Enterococcus hirae*	2216	0
4	ISLpl1	IS30		*Lactobacillus plantarum*	2028	0
5	ISPp1	IS30		*Pediococcus pentosaceus*	1925	0
6	ISLpl3	IS5	IS427	*Lactobacillus plantarum*	1643	0
7	ISLhe30	IS30		*Lactobacillus helveticus*	1473	0
8	ISLsa1	IS30		*Lactobacillus sakei*	1372	0
9	ISLpl2	IS3	IS150	*Lactobacillus plantarum*	61.9	0.000006
10	ISBame1	IS256		*Bacillus megaterium*	58	0.0001

TableS9. Match between antibiotic resistance gene search using KofamKOALA (KEGG Orthology) web servers and phenotypic antibiotic resistance results

Antibiotic group	Antibiotic	Inhibition zone diameter/status	Antibiotic Resistance Genes		
			KofamKOALA(ver. 2023-06-07, KEGG release 106.0)		
			Gene	Product	E-value
β-Lactams	Penicillin G (10U)	19.9 (S)	*pbp2A*	penicillin-binding protein 2A [EC:2.4.1.129 3.4.16.4]	2.70E-287
			penP	beta-lactamase class A [EC:3.5.2.6]	8.40E-51
	Ampicillin (10μg)	34.70 (S)	*abcA*	ATP-binding cassette, subfamily B, bacterial AbcA/BmrA [EC:7.6.2.2]	4.30E-204
			oppA	oligopeptide transport system substrate-binding protein	7.00E-141
	Amoxicillin (20μg)	34.29 (S)	*oppB*	oligopeptide transport system permease protein	6.20E-123
			oppC	oligopeptide transport system permease protein	1.70E-113
	Cefotaxime (30μg)	32.93 (S)	*oppD*	oligopeptide transport system ATP-binding protein	1.20E-197
			oppF	oligopeptide transport system ATP-binding protein	2.40E-161
Aminoglycosides	Kanamycin (30μg)	≤13(R)	-	-	-
	Gentamicin (10μg)	16.98 (S)	-	-	-
Macrolides	Erythromycin (15μg)	25.48 (S)	-	-	-
Tetracyclines	Tetracycline (30μg)	20.99 (S)	*tetM*	ribosomal protection tetracycline resistance protein	2.10E-276
	Minocycline (30μg)	24.00mm (S)	-	-	-
4-quinolones	Ciprofloxacin (5μg)	≤15 (R)	-	-	-
	Norfloxacin (10μg)	≤12 (R)	-	-	-
	Enrofloxacin (10μg)	14.18 (I)	-	-	-
Glycopeptides	Vancomycin (30μg)	≤14 (R)	*vanX*	zinc D-Ala-D-Ala dipeptidase [EC:3.4.13.22]	3.90E-61
			vanY	zinc D-Ala-D-Ala carboxypeptidase [EC:3.4.17.14]	2.60E-50
			alr	alanine racemase [EC:5.1.1.1]	3.30E-129
			ddl	D-alanine-D-alanine ligase [EC:6.3.2.4]	3.90E-109
			mraY	phospho-N-acetylmuramoyl-pentapeptide-transferase [EC:2.7.8.13]	3.50E-119
			murF	UDP-N-acetylmuramoyl-tripeptide--D-alanyl-D-alanine ligase [EC:6.3.2.10]	1.40E-147
			murG	UDP-N-acetylglucosamine--N-acetylmuramyl-(pentapeptide) pyrophosphoryl-undecaprenol N-acetylglucosamine transferase [EC:2.4.1.227]	2.10E-128
sulfa drugs	Sulffurazole (300μg)	≤12 (R)	-	-	-
Lincomycin	Lincomycin	Not tested	*lmrB*	MFS transporter, DHA2 family, lincomycin resistance protein	4.60E-139

Table S10. Horizontal gene transfer screenING for antibiotic resistance genes

Antibiotic	Gene	Protein BLAST result	Accession	Identity	E-Value
β-Lactams	*pbp2A*	PBP1A family penicillin-binding protein [*Lactiplantibacillus plantarum*]	QSE53449.1	99%	0.0
	penP	serine hydrolase [*Lactiplantibacillus plantarum*]	WP_011101017.1	100.00%	0.0
Tetracycline	*tetM*	TetM/TetW/TetO/TetS family tetracycline resistance ribosomal protection protein [*Lactiplantibacillus plantarum*]	WP_027822280.1	100.00%	0.0
Lincomycin	*lmrB*	DHA2 family efflux MFS transporter permease subunit [*Lactiplantibacillus plantarum*]	WP_015640444.1	100.00%	0.0
Vancomycin	*vanX*	M15 family metallopeptidase [*Lactiplantibacillus plantarum*]	WP_015825207.1	100.00%	3e-134
	vanY	M15 family metallopeptidase [*Lactiplantibacillus plantarum*]	WP_016511489.1	100.00%	3e-180
	alr	alanine racemase [*Lactiplantibacillus plantarum*]	GJI53677.1	100.00%	0.0
	ddl	D-alanine--D-alanine ligase [*Lactiplantibacillus plantarum* ZJ316]	AGE39858.1	99.73%	0.0
	mraY	phospho-N-acetylmuramoyl-pentapeptide-transferase [*Lactiplantibacillus plantarum*]	WP_003640859.1	100.00%	0.0
	murF	UDP-N-acetylmuramoyl-tripeptide--D-alanyl-D-alanine ligase [*Lactiplantibacillus plantarum*]	WP_003643844.1	99.78%	0.0
	murG	undecaprenyldiphospho-muramoylpentapeptide beta-N-acetylglucosaminyltransferase [*Lactiplantibacillus plantarum*]	WP_003640857.1	100.00%	0.0
Efflux pumps	*efrB*	ABC transporter ATP-binding protein [*Lactiplantibacillus plantarum*]	WP_111443160.1	99.84%	0.0
	efrA	ABC transporter ATP-binding protein [*Lactiplantibacillus plantarum*]	WP_003641422.1	100.00%	0.0
	mdlB	Lipid A export ATP-binding/permease proteinMsbA [*Lactiplantibacillus plantarum*]	KZU11536.1	99.83%	0.0
	mdlA	ABC transporter transmembrane domain-containing protein [*Lactiplantibacillus plantarum*]	WP_016511510.1	100.00%	0.0
	patB	ABC transporter ATP-binding protein [*Lactiplantibacillus plantarum*]	WP_249391052.1	99.83%	0.0
	patA	ABC transporter ATP-binding protein [*Lactiplantibacillus plantarum*]	WP_027822837.1	100.00%	0.0
	abcA	ABC transporter ATP-binding protein [*Lactiplantibacillus plantarum*]	WP_054519139.1	100.00%	0.0
	oppA	peptide ABC transporter substrate-binding protein [*Lactiplantibacillus plantarum*]	WP_072533228.1	99.81%	0.0
	oppB	ABC transporter permease [*Lactiplantibacillus plantarum*]	WP_072536085.1	99.68%	0.0
	oppC	ABC transporter permease [*Lactiplantibacillus plantarum*]	WP_259610052.1	99.71%	0.0
	oppD	ABC transporter ATP-binding protein [*Lactiplantibacillus plantarum*]	WP_260390669.1	100.00%	0.0
	oppF	ATP-binding cassette domain-containing protein [*Lactiplantibacillus plantarum*]	WP_131072774.1	99%	0.0
	blt	tetracycline resistance MFS efflux pump [*Lactiplantibacillus plantarum*]	WP_003641027.1	100%	0.0
	mdtG	MFS transporter [*Lactiplantibacillus plantarum*]	WP_211758286.1	100%	0.0

Table S11. Carbohydrate metabolism genes annotated by KEGG orthology

Glycolysis / Gluconeogenesis

#	Symbol	KEGG Entry	Definition	Copy Number
1	E1.1.1.1, *adh*	K00001	alcohol dehydrogenase [EC:1.1.1.1]	3
2	*adhE*	K04072	acetaldehyde dehydrogenase / alcohol dehydrogenase [EC:1.2.1.10 1.1.1.1]	2
**3**	**FBA, *fbaA***	**K01624**	**fructose-bisphosphate aldolase, class II [EC:4.1.2.13]**	**1**
4	LDH, ldh	K00016	L-lactate dehydrogenase [EC:1.1.1.27]	6
5	E3.2.1.86B, *bglA*	K01223	6-phospho-beta-glucosidase [EC:3.2.1.86]	7
6	PGAM, *gpmA*	K01834	2,3-bisphosphoglycerate-dependent phosphoglycerate mutase [EC:5.4.2.11]	2
7	*pgm*	K01835	phosphoglucomutase [EC:5.4.2.2]	1
8	GAPDH, *gapA*	K00134	glyceraldehyde 3-phosphate dehydrogenase (phosphorylating) [EC:1.2.1.12]	1
9	PGK, *pgk*	K00927	phosphoglycerate kinase [EC:2.7.2.3]	1
10	TPI, *tpiA*	K01803	triosephosphate isomerase (TIM) [EC:5.3.1.1]	1
11	ENO, *eno*	K01689	enolase [EC:4.2.1.11]	2
**12**	***galM*, GALM**	**K01785**	**aldose 1-epimerase [EC:5.1.3.3]**	**3**
13	*crr*	K02777	sugar PTS system EIIA component [EC:2.7.1.-]	2
14	*glk*	K25026	glucokinase [EC:2.7.1.2]	1
15	PK, *pyk*	K00873	pyruvate kinase [EC:2.7.1.40]	1
**16**	***pfkA*, PFK**	**K00850**	**6-phosphofructokinase 1 [EC:2.7.1.11]**	**1**
17	*pps*, *ppsA*	K01007	pyruvate, water dikinase [EC:2.7.9.2]	1
18	DLD, *lpd*, *pdhD*	K00382	dihydrolipoamide dehydrogenase [EC:1.8.1.4]	1
19	DLAT, *aceF*, *pdhC*	K00627	pyruvate dehydrogenase E2 component (dihydrolipoamide acetyltransferase) [EC:2.3.1.12]	1
20	PDHB, *pdhB*	K00162	pyruvate dehydrogenase E1 component beta subunit [EC:1.2.4.1]	1
21	PDHA, *pdhA*	K00161	pyruvate dehydrogenase E1 component alpha subunit [EC:1.2.4.1]	1
**22**	**GPI, *pgi***	**K01810**	**glucose-6-phosphate isomerase [EC:5.3.1.9]**	**1**
23	E4.1.1.49, *pckA*	K01610	phosphoenolpyruvate carboxykinase (ATP) [EC:4.1.1.49]	1

Citrate cycle (TCA cycle)

#	Symbol	KEGG Entry	Definition	Copy Number
1	E4.2.1.2B, *fumC*, FH	K01679	fumarate hydratase, class II [EC:4.2.1.2]	1
2	PC, *pyc*	K01958	pyruvate carboxylase [EC:6.4.1.1]	1
3	DLD, *lpd*, *pdhD*	K00382	dihydrolipoamide dehydrogenase [EC:1.8.1.4]	1
4	DLAT, *aceF*, *pdhC*	K00627	pyruvate dehydrogenase E2 component (dihydrolipoamide acetyltransferase) [EC:2.3.1.12]	1
5	PDHB, *pdhB*	K00162	pyruvate dehydrogenase E1 component beta subunit [EC:1.2.4.1]	1
6	PDHA, *pdhA*	K00161	pyruvate dehydrogenase E1 component alpha subunit [EC:1.2.4.1]	1
7	E4.1.1.49, *pckA*	K01610	phosphoenolpyruvate carboxykinase (ATP) [EC:4.1.1.49]	1

Pentose phosphate pathway

#	Symbol	KEGG Entry	Definition	Copy Numbe
**1**	**FBA, *fbaA***	**K01624**	**fructose-bisphosphate aldolase, class II [EC:4.1.2.13]**	**1**
2	*rpiA*	K01807	ribose 5-phosphate isomerase A [EC:5.3.1.6]	2
3	PRPS, *prsA*	K00948	ribose-phosphate pyrophosphokinase [EC:2.7.6.1]	2
4	*eda*	K01625	2-dehydro-3-deoxyphosphogluconate aldolase / (4S)-4-hydroxy-2-oxoglutarate aldolase [EC:4.1.2.14 4.1.3.42]	1
5	*pgm*	K01835	phosphoglucomutase [EC:5.4.2.2]	1

6	E2.2.1.1, *tktA*, *tktB*	K00615	transketolase [EC:2.2.1.1]	1
7	*gntK*	K25031	gluconokinase [EC:2.7.1.12]	1
8	PGD, *gnd*, *gntZ*	K00033	6-phosphogluconate dehydrogenase [EC:1.1.1.44 1.1.1.343]	2
**9**	***rpe*, RPE**	**K01783**	**ribulose-phosphate 3-epimerase [EC:5.1.3.1]**	**1**
**10**	***pfkA*, PFK**	**K00850**	**6-phosphofructokinase 1 [EC:2.7.1.11]**	**1**
11	*pgl*	K07404	6-phosphogluconolactonase [EC:3.1.1.31]	1
12	*rbsK*, RBKS	K00852	ribokinase [EC:2.7.1.15]	2
**13**	**GPI, *pgi***	**K01810**	**glucose-6-phosphate isomerase [EC:5.3.1.9]**	**1**
14	*xfp*, *xpk*	K01621	xylulose-5-phosphate/fructose-6-phosphate phosphoketolase [EC:4.1.2.9 4.1.2.22]	1
15	G6PD, *zwf*	K00036	glucose-6-phosphate 1-dehydrogenase [EC:1.1.1.49 1.1.1.363]	1
Pentose and glucuronate interconversions				
#	Symbol	KEGG Entry	Definition	Copy Number
**1**	**UGP2, *galU*, *galF***	**K00963**	**UTP--glucose-1-phosphate uridylyltransferase [EC:2.7.7.9]**	**1**
**2**	***rpe*, RPE**	**K01783**	**ribulose-phosphate 3-epimerase [EC:5.1.3.1]**	**1**
3	*tarI*	K21030	D-ribitol-5-phosphate cytidylyltransferase [EC:2.7.7.40]	1
4	E1.1.1.137	K21680	ribulose-5-phosphate 2-dehydrogenase [EC:1.1.1.137]	1
Fructose and mannose metabolism				
#	Symbol	KEGG Entry	Definition	Copy Number
1	DAK, TKFC	K00863	triose/dihydroxyacetone kinase / FAD-AMP lyase (cyclizing) [EC:2.7.1.28 2.7.1.29 4.6.1.15]	1
2	E2.7.1.4, *scrK*	K00847	fructokinase [EC:2.7.1.4]	2
3	*mtlA*, *cmtA*	K02800	mannitol PTS system EIICBA or EIICB component [EC:2.7.1.197]	1
4	*cmtB*	K02798	mannitol PTS system EIIA component [EC:2.7.1.197]	1
5	*mtlD*	K00009	mannitol-1-phosphate 5-dehydrogenase [EC:1.1.1.17]	1
**6**	**FBA, *fbaA***	**K01624**	**fructose-bisphosphate aldolase, class II [EC:4.1.2.13]**	**1**
7	*manX*	K02794	mannose PTS system EIIAB component [EC:2.7.1.191]	1
8	*manY*	K02795	mannose PTS system EIIC component	1
9	*manZ*	K02796	mannose PTS system EIID component	1
10	*manXa*	K02793	mannose PTS system EIIA component [EC:2.7.1.191]	2
11	*fruAb*	K02769	fructose PTS system EIIB component [EC:2.7.1.202]	2
12	TPI, *tpiA*	K01803	triosephosphate isomerase (TIM) [EC:5.3.1.1]	1
**13**	***pfkA*, PFK**	**K00850**	**6-phosphofructokinase 1 [EC:2.7.1.11]**	**1**
14	*fruK*	K00882	1-phosphofructokinase [EC:2.7.1.56]	1
15	*fruA*	K02770	fructose PTS system EIIBC or EIIC component [EC:2.7.1.202]	2
16	*srlB*	K02781	glucitol/sorbitol PTS system EIIA component [EC:2.7.1.198]	2
17	*manA*, MPI	K01809	mannose-6-phosphate isomerase [EC:5.3.1.8]	1
18	*fruB*	K02768	fructose PTS system EIIA component [EC:2.7.1.202]	1
19	*srlE*	K02782	glucitol/sorbitol PTS system EIIB component [EC:2.7.1.198]	1
20	*srlA*	K02783	glucitol/sorbitol PTS system EIIC component	1
21	*srlD*	K00068	sorbitol-6-phosphate 2-dehydrogenase [EC:1.1.1.140]	1
Galactose metabolism				
#	Symbol	KEGG Entry	Definition	Copy Number
1	*malZ*	K01187	alpha-glucosidase [EC:3.2.1.20]	1
2	E3.2.1.22B, galA, rafA	K07407	alpha-galactosidase [EC:3.2.1.22]	3

3	INV, *sacA*	K01193	beta-fructofuranosidase [EC:3.2.1.26]	1
4	IMA, *malL*	K01182	oligo-1,6-glucosidase [EC:3.2.1.10]	2
5	*galE*, GALE	K01784	UDP-glucose 4-epimerase [EC:5.1.3.2]	3
6	UGP2, *galU*, *galF*	K00963	UTP--glucose-1-phosphate uridylyltransferase [EC:2.7.7.9]	1
7	*pgm*	K01835	phosphoglucomutase [EC:5.4.2.2]	1
8	*galM*, GALM	K01785	aldose 1-epimerase [EC:5.1.3.3]	3
9	*glk*	K25026	glucokinase [EC:2.7.1.2]	1
**10**	***pfkA*, PFK**	**K00850**	**6-phosphofructokinase 1 [EC:2.7.1.11]**	**1**
11	*agaF*	K02744	N-acetylgalactosamine PTS system EIIA component [EC:2.7.1.-]	1
12	*agaD*	K10986	galactosamine PTS system EIID component	1
13	*agaC*	K10985	galactosamine PTS system EIIC component	1
14	*agaB*	K10984	galactosamine PTS system EIIB component [EC:2.7.1.-]	1
15	*bgaB*, *lacA*	K12308	beta-galactosidase [EC:3.2.1.23]	2
16	*galT*, GALT	K00965	UDPglucose--hexose-1-phosphate uridylyltransferase [EC:2.7.7.12]	1
17	*galK*	K00849	galactokinase [EC:2.7.1.6]	1
18	*lacZ*	K01190	beta-galactosidase [EC:3.2.1.23]	2

Ascorbate and aldarate metabolism

#	Symbol	KEGG Entry	Definition	Copy Number
1	*ulaC*, *sgaA*	K02821	ascorbate PTS system EIIA or EIIAB component [EC:2.7.1.194]	1
2	*ulaB*, *sgaB*	K02822	ascorbate PTS system EIIB component [EC:2.7.1.194]	1
3	*ulaA*, *sgaT*	K03475	ascorbate PTS system EIIC component	1

Starch and sucrose metabolism

#	Symbol	KEGG Entry	Definition	Copy Number
1	GBE1, *glgB*	K00700	1,4-alpha-glucan branching enzyme [EC:2.4.1.18]	2
2	*glgC*	K00975	glucose-1-phosphate adenylyltransferase [EC:2.7.7.27]	5
3	*glgA*	K00703	starch synthase [EC:2.4.1.21]	3
4	PYG, *glgP*	K00688	glycogen phosphorylase [EC:2.4.1.1]	3
5	*pgmB*	K01838	beta-phosphoglucomutase [EC:5.4.2.6]	2
6	malZ	K01187	alpha-glucosidase [EC:3.2.1.20]	1
7	AMY, *amyA*, *malS*	K01176	alpha-amylase [EC:3.2.1.1]	1
8	*mapA*	K00691	maltose phosphorylase [EC:2.4.1.8]	2
9	E2.7.1.4, *scrK*	K00847	fructokinase [EC:2.7.1.4]	2
10	*scrA*, *sacP*, *sacX*, *ptsS*	K02810	sucrose PTS system EIIBCA or EIIBC component [EC:2.7.1.211]	2
11	INV, *sacA*	K01193	beta-fructofuranosidase [EC:3.2.1.26]	1
12	IMA, *malL*	K01182	oligo-1,6-glucosidase [EC:3.2.1.10]	2
13	*treC*	K01226	trehalose-6-phosphate hydrolase [EC:3.2.1.93]	1
14	*celB*, *chbC*	K02761	cellobiose PTS system EIIC component	12
15	E3.2.1.86B, *bglA*	K01223	6-phospho-beta-glucosidase [EC:3.2.1.86]	7
16	UGP2, *galU*, *galF*	K00963	UTP--glucose-1-phosphate uridylyltransferase [EC:2.7.7.9]	1
17	*pgm*	K01835	phosphoglucomutase [EC:5.4.2.2]	1
18	*crr*	K02777	sugar PTS system EIIA component [EC:2.7.1.-]	2
19	*celC*, *chbA*	K02759	cellobiose PTS system EIIA component [EC:2.7.1.196 2.7.1.205]	3
20	*celA*, *chbB*	K02760	cellobiose PTS system EIIB component [EC:2.7.1.196 2.7.1.205]	4

21	*glk*	K25026	glucokinase [EC:2.7.1.2]	1
**22**	**GPI, *pgi***	**K01810**	**glucose-6-phosphate isomerase [EC:5.3.1.9]**	**1**
23	*cd*, *ma*, *nplT*	K01208	cyclomaltodextrinase / maltogenic alpha-amylase / neopullulanase [EC:3.2.1.54 3.2.1.133 3.2.1.135]	1

Amino sugar and nucleotide sugar metabolism

#	Symbol	KEGG Entry	Definition	Copy Number
1	*glgC*	K00975	glucose-1-phosphate adenylyltransferase [EC:2.7.7.27]	5
2	E2.7.1.4, *scrK*	K00847	fructokinase [EC:2.7.1.4]	2
3	*nagB*, GNPDA	K02564	glucosamine-6-phosphate deaminase [EC:3.5.99.6]	1
4	*glmU*	K04042	bifunctional UDP-N-acetylglucosamine pyrophosphorylase / glucosamine-1-phosphate N-acetyltransferase [EC:2.7.7.23 2.3.1.157]	1
5	*murA*	K00790	UDP-N-acetylglucosamine 1-carboxyvinyltransferase [EC:2.5.1.7]	2
6	*nagA*, AMDHD2	K01443	N-acetylglucosamine-6-phosphate deacetylase [EC:3.5.1.25]	1
7	*manX*	K02794	mannose PTS system EIIAB component [EC:2.7.1.191]	1
8	*manY*	K02795	mannose PTS system EIIC component	1
9	*manZ*	K02796	mannose PTS system EIID component	1
10	*manXa*	K02793	mannose PTS system EIIA component [EC:2.7.1.191]	2
11	*galE*, GALE	K01784	UDP-glucose 4-epimerase [EC:5.1.3.2]	3
12	UGP2, *galU*, *galF*	K00963	UTP--glucose-1-phosphate uridylyltransferase [EC:2.7.7.9]	1
13	*pgm*	K01835	phosphoglucomutase [EC:5.4.2.2]	1
14	*murB*	K00075	UDP-N-acetylmuramate dehydrogenase [EC:1.3.1.98]	1
15	*glmM*	K03431	phosphoglucosamine mutase [EC:5.4.2.10]	1
16	*glmS*, GFPT	K00820	glutamine---fructose-6-phosphate transaminase (isomerizing) [EC:2.6.1.16]	1
17	*mupP*	K22292	N-acetyl-D-muramate 6-phosphate phosphatase [EC:3.1.3.105]	1
18	*crr*	K02777	sugar PTS system EIIA component [EC:2.7.1.-]	2
19	*wecB*	K01791	UDP-N-acetylglucosamine 2-epimerase (non-hydrolysing) [EC:5.1.3.14]	1
20	*glk*	K25026	glucokinase [EC:2.7.1.2]	1
21	*manA*, MPI	K01809	mannose-6-phosphate isomerase [EC:5.3.1.8]	1
**22**	**GPI, *pgi***	**K01810**	**glucose-6-phosphate isomerase [EC:5.3.1.9]**	**1**
23	*nagE*	K02804	N-acetylglucosamine PTS system EIICBA or EIICB component [EC:2.7.1.193]	2
24	*galT*, GALT	K00965	UDPglucose--hexose-1-phosphate uridylyltransferase [EC:2.7.7.12]	1
25	*galK*	K00849	galactokinase [EC:2.7.1.6]	1
26	*murQ*	K07106	N-acetylmuramic acid 6-phosphate etherase [EC:4.2.1.126]	1
27	*nanE*	K01788	N-acylglucosamine-6-phosphate 2-epimerase [EC:5.1.3.9]	1

Pyruvate metabolism

#	Symbol	KEGG Entry	Definition	Copy Number
1	E1.1.1.1, adh	K00001	alcohol dehydrogenase [EC:1.1.1.1]	3
2	*larA*	K22373	lactate racemase [EC:5.1.2.1]	1
3	*ackA*	K00925	acetate kinase [EC:2.7.2.1]	3
4	*adhE*	K04072	acetaldehyde dehydrogenase / alcohol dehydrogenase [EC:1.2.1.10 1.1.1.1]	2
5	LDH, *ldh*	K00016	L-lactate dehydrogenase [EC:1.1.1.27]	6
6	*accB*, *bccP*	K02160	acetyl-CoA carboxylase biotin carboxyl carrier protein	3
7	*accC*	K01961	acetyl-CoA carboxylase, biotin carboxylase subunit [EC:6.4.1.2 6.3.4.14]	2
8	*accD*	K01963	acetyl-CoA carboxylase carboxyl transferase subunit beta [EC:6.4.1.2 2.1.3.15]	2

9	*accA*	K01962	acetyl-CoA carboxylase carboxyl transferase subunit alpha [EC:6.4.1.2 2.1.3.15]	2
10	*pta*	K00625	phosphate acetyltransferase [EC:2.3.1.8]	1
11	*spxB*, *poxL*	K00158	pyruvate oxidase [EC:1.2.3.3]	5
12	*ldhA*	K03778	D-lactate dehydrogenase [EC:1.1.1.28]	2
13	ME2, *sfcA*, *maeA*	K00027	malate dehydrogenase (oxaloacetate-decarboxylating) [EC:1.1.1.38]	1
14	E4.2.1.2B, *fumC*, FH	K01679	fumarate hydratase, class II [EC:4.2.1.2]	1
15	*mleA*, *mleS*	K22212	malolactic enzyme [EC:4.1.1.101]	1
16	*acyP*	K01512	acylphosphatase [EC:3.6.1.7]	1
17	PK, *pyk*	K00873	pyruvate kinase [EC:2.7.1.40]	1
18	*pps*, *ppsA*	K01007	pyruvate, water dikinase [EC:2.7.9.2]	1
19	PC, *pyc*	K01958	pyruvate carboxylase [EC:6.4.1.1]	1
20	DLD, *lpd*, *pdhD*	K00382	dihydrolipoamide dehydrogenase [EC:1.8.1.4]	1
21	DLAT, *aceF*, *pdhC*	K00627	pyruvate dehydrogenase E2 component (dihydrolipoamide acetyltransferase) [EC:2.3.1.12]	1
22	PDHB, *pdhB*	K00162	pyruvate dehydrogenase E1 component beta subunit [EC:1.2.4.1]	1
23	PDHA, *pdhA*	K00161	pyruvate dehydrogenase E1 component alpha subunit [EC:1.2.4.1]	1
24	E2.3.1.54, *pflD*	K00656	formate C-acetyltransferase [EC:2.3.1.54]	1
25	E4.1.1.49, *pckA*	K01610	phosphoenolpyruvate carboxykinase (ATP) [EC:4.1.1.49]	1

Glyoxylate and dicarboxylate metabolism

#	Symbol	KEGG Entry	Definition	Copy Number
1	*glcD*	K00104	glycolate oxidase [EC:1.1.3.15]	1
2	*gcvH*, GCSH	K02437	glycine cleavage system H protein	2
3	*eda*	K01625	2-dehydro-3-deoxyphosphogluconate aldolase / (4S)-4-hydroxy-2-oxoglutarate aldolase [EC:4.1.2.14 4.1.3.42]	1
4	*ttdB*	K03780	L(+)-tartrate dehydratase beta subunit [EC:4.2.1.32]	1
5	*ttdA*	K03779	L(+)-tartrate dehydratase alpha subunit [EC:4.2.1.32]	1
6	*glnA*, GLUL	K01915	glutamine synthetase [EC:6.3.1.2]	1
7	*gph*	K01091	phosphoglycolate phosphatase [EC:3.1.3.18]	1
8	DLD, *lpd*, *pdhD*	K00382	dihydrolipoamide dehydrogenase [EC:1.8.1.4]	1
9	*glyA*, SHMT	K00600	glycine hydroxymethyltransferase [EC:2.1.2.1]	1
10	*glxK*, *garK*	K00865	glycerate 2-kinase [EC:2.7.1.165]	1
11	*katE*, CAT, *catB*, *srpA*	K03781	catalase [EC:1.11.1.6]	1

Propanoate metabolism

#	Symbol	KEGG Entry	Definition	Copy Number
1	*ackA*	K00925	acetate kinase [EC:2.7.2.1]	3
2	LDH, *ldh*	K00016	L-lactate dehydrogenase [EC:1.1.1.27]	6
3	*accB*, *bccP*	K02160	acetyl-CoA carboxylase biotin carboxyl carrier protein	3
4	*accC*	K01961	acetyl-CoA carboxylase, biotin carboxylase subunit [EC:6.4.1.2 6.3.4.14]	2
5	*accD*	K01963	acetyl-CoA carboxylase carboxyl transferase subunit beta [EC:6.4.1.2 2.1.3.15]	2
6	*accA*	K01962	acetyl-CoA carboxylase carboxyl transferase subunit alpha [EC:6.4.1.2 2.1.3.15]	2
7	*pta*	K00625	phosphate acetyltransferase [EC:2.3.1.8]	1
8	DLD, *lpd*, *pdhD*	K00382	dihydrolipoamide dehydrogenase [EC:1.8.1.4]	1
9	*dhaT*	K00086	1,3-propanediol dehydrogenase [EC:1.1.1.202]	1
10	E2.3.1.54, *pflD*	K00656	formate C-acetyltransferase [EC:2.3.1.54]	1

C5-Branched dibasic acid metabolism				
#	Symbol	KEGG Entry	Definition	Copy Number
1	E2.2.1.6L, *ilvB*, *ilvG*, *ilvI*	K01652	acetolactate synthase I/II/III large subunit [EC:2.2.1.6]	1
2	*alsD*, *budA*, *aldC*	K01575	acetolactate decarboxylase [EC:4.1.1.5]	1

Inositol phosphate metabolism				
#	Symbol	KEGG Entry	Definition	Copy Number
1	TPI, *tpiA*	K01803	triosephosphate isomerase (TIM) [EC:5.3.1.1]	1
2	E3.1.3.25, IMPA, *suhB*	K01092	myo-inositol-1(or 4)-monophosphatase [EC:3.1.3.25]	1
3	*iolU*	K22230	scyllo-inositol 2-dehydrogenase (NADP+) [EC:1.1.1.-]	3
4	*iolI*	K06606	2-keto-myo-inositol isomerase [EC:5.3.99.11]	2
5	*iolG*	K00010	myo-inositol 2-dehydrogenase / D-chiro-inositol 1-dehydrogenase [EC:1.1.1.18 1.1.1.369]	7
6	*iolE*	K03335	inosose dehydratase [EC:4.2.1.44]	1

Butanoate metabolism				
#	Symbol	KEGG Entry	Definition	Copy Number
1	*adc*	K01574	acetoacetate decarboxylase [EC:4.1.1.4]	1
2	*adhE*	K04072	acetaldehyde dehydrogenase / alcohol dehydrogenase [EC:1.2.1.10 1.1.1.1]	2
3	E2.2.1.6L, *ilvB*, *ilvG*, *ilvI*	K01652	acetolactate synthase I/II/III large subunit [EC:2.2.1.6]	1
4	*alsD*, *budA*, *aldC*	K01575	acetolactate decarboxylase [EC:4.1.1.5]	1
5	HMGCS	K01641	hydroxymethylglutaryl-CoA synthase [EC:2.3.3.10]	1
6	*gabD*	K00135	succinate-semialdehyde dehydrogenase / glutarate-semialdehyde dehydrogenase [EC:1.2.1.16 1.2.1.79 1.2.1.20]	1
7	E2.3.1.54, *pflD*	K00656	formate C-acetyltransferase [EC:2.3.1.54]	1
8	E4.1.1.15, *gadB*, *gadA*, GAD	K01580	glutamate decarboxylase [EC:4.1.1.15]	1

Table S12. KEGG (BlastKOALA) orthology search results for ABC transporters

#	Symbol	KEGG Entry	Definition	Copy Number
**1**	***oppA, mppA***	**K15580**	**oligopeptide transport system substrate-binding protein**	**8**
2	*cbiO*	K02006	cobalt/nickel transport system ATP-binding protein	2
3	*cbiM*	K02007	cobalt/nickel transport system permease protein	1
4	*ecfT*	K16785	energy-coupling factor transport system permease protein	3
5	*cycB*, *ganO*, *mdxE*	K15770	arabinogalactan oligomer / maltooligosaccharide transport system substrate-binding protein	1
6	*ganP*, *mdxF*	K15771	arabinogalactan oligomer / maltooligosaccharide transport system permease protein	1
7	*ganQ*, *mdxG*	K15772	arabinogalactan oligomer / maltooligosaccharide transport system permease protein	1
8	*msmX*, *msmK*, *malK*, *sugC, ggtA*, *msiK*	K10112	multiple sugar transport system ATP-binding protein [EC:7.5.2.-]	1
9	*msr*, *vmlR*	K18231	macrolide transport system ATP-binding/permease protein	1
10	*potD*	K11069	spermidine/putrescine transport system substrate-binding protein	1
11	*potC*	K11070	spermidine/putrescine transport system permease protein	1
12	*potB*	K11071	spermidine/putrescine transport system permease protein	1
13	*potA*	K11072	spermidine/putrescine transport system ATP-binding protein [EC:7.6.2.11]	1
14	*ytrB*	K16921	acetoin utilization transport system ATP-binding protein	2
15	*ytrC_D*	K16919	acetoin utilization transport system permease protein	1
16	*bioY*	K03523	biotin transport system substrate-specific component	1
17	*metQ*	K02073	D-methionine transport system substrate-binding protein	3
18	*metN*	K02071	D-methionine transport system ATP-binding protein	3
19	*metI*	K02072	D-methionine transport system permease protein	3
20	*tagG*	K09692	teichoic acid transport system permease protein	1
21	*tagH*	K09693	teichoic acid transport system ATP-binding protein [EC:7.5.2.4]	1
22	*opuC*	K05845	osmoprotectant transport system substrate-binding protein	2
23	*opuA*	K05847	osmoprotectant transport system ATP-binding protein [EC:7.6.2.9]	2
24	*blpA*, *lagD*	K20344	ATP-binding cassette, subfamily C, bacteriocin exporter	2
25	*phnE*	K02042	phosphonate transport system permease protein	2
26	*phnC*	K02041	phosphonate transport system ATP-binding protein [EC:7.3.2.2]	1
27	*phnD*	K02044	phosphonate transport system substrate-binding protein	1
28	*pstS*	K02040	phosphate transport system substrate-binding protein	2
29	*pstC*	K02037	phosphate transport system permease protein	1
30	*pstA*	K02038	phosphate transport system permease protein	1
31	*pstB*	K02036	phosphate transport system ATP-binding protein [EC:7.3.2.1]	2
32	*glnH*	K10036	glutamine transport system substrate-binding protein	2
33	*glnQ*	K10038	glutamine transport system ATP-binding protein [EC:7.4.2.1]	3
34	*peb1A*, *glnH*	K10039	aspartate/glutamate/glutamine transport system substrate-binding protein	1
35	*peb1B*, *glnP*, *glnM*	K10040	aspartate/glutamate/glutamine transport system permease protein	2
36	*ecfA1*	K16786	energy-coupling factor transport system ATP-binding protein [EC:7.-.-.-]	1
37	*ecfA2*	K16787	energy-coupling factor transport system ATP-binding protein [EC:7.-.-.-]	1
38	*mntA*	K19973	manganese transport system ATP-binding protein [EC:7.2.2.5]	1
39	*mntB*	K19976	manganese transport system permease protein	1
40	*mntC*	K19975	manganese transport system substrate-binding protein	1
41	*cydD*	K16013	ATP-binding cassette, subfamily C, bacterial CydD	1
42	*cydC*	K16012	ATP-binding cassette, subfamily C, bacterial CydC	1

**43**	***oppB***	**K15581**	**oligopeptide transport system permease protein**	**2**
**44**	***oppC***	**K15582**	**oligopeptide transport system permease protein**	**2**
**45**	***oppD***	**K15583**	**oligopeptide transport system ATP-binding protein**	**2**
**46**	***oppF***	**K10823**	**oligopeptide transport system ATP-binding protein**	**2**
47	*ugpC*	K05816	sn-glycerol 3-phosphate transport system ATP-binding protein [EC:7.6.2.10]	1
48	*ugpA*	K05814	sn-glycerol 3-phosphate transport system permease protein	1
49	*ugpE*	K05815	sn-glycerol 3-phosphate transport system permease protein	2
50	*ugpB*	K05813	sn-glycerol 3-phosphate transport system substrate-binding protein	1
51	*opuBD*	K05846	osmoprotectant transport system permease protein	2
52	*afuB*, *fbpB*	K02011	iron(III) transport system permease protein	1
53	*afuA*, *fbpA*	K02012	iron(III) transport system substrate-binding protein	1
54	*afuC*, *fbpC*	K02010	iron(III) transport system ATP-binding protein [EC:7.2.2.7]	1
55	*artR*, *artM*	K23060	arginine/lysine/histidine transport system ATP-binding protein [EC:7.4.2.1]	1
56	*artQ*	K17077	arginine/lysine/histidine transport system permease protein	1
**57**	***efrB*, *efrF***	**K18888**	**ATP-binding cassette, subfamily B, multidrug efflux pump**	**1**
**58**	***efrA*, *efrE***	**K18887**	**ATP-binding cassette, subfamily B, multidrug efflux pump**	**1**
**59**	***abcA*, *bmrA***	**K18104**	**ATP-binding cassette, subfamily B, bacterial AbcA/BmrA [EC:7.6.2.2]**	**2**
**60**	***mdlB*, *smdB***	**K18890**	**ATP-binding cassette, subfamily B, multidrug efflux pump**	**1**
**61**	***mdlA*, *smdA***	**K18889**	**ATP-binding cassette, subfamily B, multidrug efflux pump**	**1**
62	*livF*	K01996	branched-chain amino acid transport system ATP-binding protein	1
63	*livG*	K01995	branched-chain amino acid transport system ATP-binding protein	1
64	*livM*	K01998	branched-chain amino acid transport system permease protein	1
65	*livH*	K01997	branched-chain amino acid transport system permease protein	1
66	*livK*	K01999	branched-chain amino acid transport system substrate-binding protein	1
**67**	***patB*, *rscB*, *lmrC*, *satB***	**K18892**	**ATP-binding cassette, subfamily B, multidrug efflux pump**	**1**
**68**	***patA*, *rscA*, *lmrC*, *satA***	**K18891**	**ATP-binding cassette, subfamily B, multidrug efflux pump**	**1**
69	*tcyA*, *tcyJ*, *fliY*	K02424	L-cystine transport system substrate-binding protein	2
70	*tcyB*, *tcyL*	K10009	L-cystine transport system permease protein	1
71	*tcyC*, *tcyN*	K10010	L-cystine transport system ATP-binding protein [EC:7.4.2.1]	1
72	*rbsD*	K06726	D-ribose pyranase [EC:5.4.99.62]	1

.

Table S13. Phosphotransferase system (PTS) annotated by KEGG (BlastKOALA)

#	Symbol	KEGG Entry	Definition	Copy Number
1	*scrA*, *sacP*, *sacX*, *ptsS*	K02810	sucrose PTS system EIIBCA or EIIBC component [EC:2.7.1.211]	2
2	*mtlA*, *cmtA*	K02800	mannitol PTS system EIICBA or EIICB component [EC:2.7.1.197]	1
3	*cmtB*	K02798	mannitol PTS system EIIA component [EC:2.7.1.197]	1
4	*bglF*, *bglP*	K02757	beta-glucoside PTS system EIICBA component [EC:2.7.1.-]	5
5	*celB*, *chbC*	K02761	cellobiose PTS system EIIC component	12
6	*manX*	K02794	mannose PTS system EIIAB component [EC:2.7.1.191]	1
7	*manY*	K02795	mannose PTS system EIIC component	1
8	*manZ*	K02796	mannose PTS system EIID component	1
9	*manXa*	K02793	mannose PTS system EIIA component [EC:2.7.1.191]	2
10	*fruAb*	K02769	fructose PTS system EIIB component [EC:2.7.1.202]	2
11	*ulaC*, *sgaA*	K02821	ascorbate PTS system EIIA or EIIAB component [EC:2.7.1.194]	1
12	*ulaB*, *sgaB*	K02822	ascorbate PTS system EIIB component [EC:2.7.1.194]	1
13	*ulaA*, *sgaT*	K03475	ascorbate PTS system EIIC component	1
14	*crr*	K02777	sugar PTS system EIIA component [EC:2.7.1.-]	2
15	*celC*, *chbA*	K02759	cellobiose PTS system EIIA component [EC:2.7.1.196 2.7.1.205]	3
16	*ptsH*	K02784	phosphocarrier protein HPr	1
17	*ptsI*	K08483	phosphoenolpyruvate-protein phosphotransferase (PTS system enzyme I) [EC:2.7.3.9]	1
18	*celA*, *chbB*	K02760	cellobiose PTS system EIIB component [EC:2.7.1.196 2.7.1.205]	4
19	*fruK*	K00882	1-phosphofructokinase [EC:2.7.1.56]	1
20	*fruA*	K02770	fructose PTS system EIIBC or EIIC component [EC:2.7.1.202]	2
21	*srlB*	K02781	glucitol/sorbitol PTS system EIIA component [EC:2.7.1.198]	2
22	*nagE*	K02804	N-acetylglucosamine PTS system EIICBA or EIICB component [EC:2.7.1.193]	2
23	*agaF*	K02744	N-acetylgalactosamine PTS system EIIA component [EC:2.7.1.-]	1
24	*agaD*	K10986	galactosamine PTS system EIID component	1
25	*agaC*	K10985	galactosamine PTS system EIIC component	1
26	*agaB*	K10984	galactosamine PTS system EIIB component [EC:2.7.1.-]	1
27	*fruB*	K02768	fructose PTS system EIIA component [EC:2.7.1.202]	1
28	*srlE*	K02782	glucitol/sorbitol PTS system EIIB component [EC:2.7.1.198]	1
29	*srlA*	K02783	glucitol/sorbitol PTS system EIIC component	1

Table S14. The predicted biosynthetic gene clusters of secondary metabolites.

Region	Length	Total Proteins	Type	Region Position	ClusterBlast
1	11.260Kb	13	RiPP-like	374,530 - 385,789	NZ_CP035143 (374689-386840), bacteriocin, 91% of genes show similarity
2	41.170Kb	38	T3PKS	1,792,491 - 1,833,660	NZ_CP028977 (1817876-1859046), T3PKS, 97% of genes show similarity
3	20.882Kb	21	terpene	2,844,604 - 2,865,485	NZ_CP015857 (2748148-2769030), terpene, 94% of genes show similarity
4	20.706Kb	19	cyclic-lactone-autoinducer	3,071,820 - 3,092,525	NZ_KB946320 (432500-458581), lanthipeptide, 13% of genes show similarity

Table S15. The RiPP-like region elements of *Lactiplantibacillus plantarum* JS21.

Gene	Start	Stop	Strand	Description	E-Value	Percent Identity	Accession
gene0357	375687	375858	+	hypothetical protein [*Lactiplantibacillus plantarum*]	4E-29	1	WP_027822766.1
gene0358	375912	377088	-	IS256 family transposase [*Lactiplantibacillus plantarum*]	0	0.9898	WP_310581100.1
gene0359	377181	377922	-	CPBP family intramembrane glutamic endopeptidase [*Lactiplantibacillus plantarum*]	7E-166	0.9917	WP_027821503.1
gene0360	378020	378179	-	two-peptide bacteriocin plantaricin EF subunit PlnF [*Lactiplantibacillus plantarum*]	5E-29	1	WP_027822764.1
gene0361	378203	378374	-	two-peptide bacteriocin plantaricin EF subunit PlnE [*Lactiplantibacillus plantarum*]	2E-30	0.9821	WP_102115620.1
gene0362（*blpA*）	378639	379470	+	Transport/processing ATP-binding protein ComA [*Lactiplantibacillus plantarum*]	0	0.9819	OUS99406.1
gene0363（*blpA*）	379529	380789	+	peptide cleavage/export ABC transporter [*Lactiplantibacillus plantarum*]	0	0.9976	WP_234521144.1
gene0364（*blpB*）	380805	382182	+	HlyD family secretion protein [*Lactiplantibacillus plantarum*]	0	1	WP_027821501.1
gene0365	382271	382961	+	CPBP family intramembrane glutamic endopeptidase [*Lactiplantibacillus plantarum*]	3E-163	0.9956	WP_053339246.1
gene0366	383028	383697	+	CPBP family intramembrane glutamic endopeptidase [*Lactiplantibacillus plantarum*]	6E-156	1	WP_027822761.1
gene0367	383783	384464	+	CPBP family intramembrane glutamic endopeptidase [*Lactiplantibacillus plantarum*]	5E-160	1	WP_027822760.1
gene0368	384557	385232	+	CPBP family intramembrane glutamic endopeptidase [*Lactiplantibacillus plantarum*]	7E-158	1	WP_027822759.1
gene0369(*higB-1*)	385379	385670	+	type II toxin-antitoxin system RelE/ParE family toxin [*Lactiplantibacillus plantarum*]	3E-65	1	WP_027822758.1

Table S16. The T3PKS region elements of *Lactiplantibacillus plantarum* JS21.

Gene	Start	Stop	Strand	Description	E-Value	Percent Identity	Accession
gene1731(*polC*)	1792817	1797131	-	DNA polymerase III subunit alpha [*Lactiplantibacillus plantarum*]	0	1	WP_003640729.1
gene1732(*proS*)	1797624	1799334	-	proline--tRNA ligase [*Lactiplantibacillus plantarum*]	0	0.9982	WP_185936516.1
gene1733(*rseP*)	1799373	1800651	-	RIP metalloprotease RseP [*Lactiplantibacillus plantarum*]	0	0.9976	WP_172637917.1
gene1734（*cdsA*）	1800688	1801474	-	phosphatidate cytidylyltransferase [*Lactiplantibacillus plantarum*]	0	0.9962	WP_251335027.1
gene1735（*uppS*）	1801489	1802269	-	isoprenyl transferase [*Lactiplantibacillus plantarum*]	0	0.9961	WP_045351394.1
gene1736（*frr*）	1802388	1802952	-	ribosome recycling factor [*Lactiplantibacillus plantarum*]	1E-129	0.9947	WP_208664135.1
gene1737（*pyrH*）	1802953	1803676	-	UMP kinase [*Lactiplantibacillus plantarum*]	1E-173	0.9958	WP_079111798.1
gene1738（*tsf*）	1803875	1804754	-	translation elongation factor Ts [*Lactiplantibacillus plantarum*]	0	0.9966	WP_135517078.1
gene1739（*rpsB*）	1804856	1805660	-	30S ribosomal protein S2 [*Lactiplantibacillus plantarum*]	0	0.9963	WP_259689552.1
gene1740	1805884	1806607	+	HAD family hydrolase [*Lactiplantibacillus plantarum*]	3E-175	0.9958	WP_160248452.1
gene1741（*ldhA*）	1806896	1807895	-	D-2-hydroxyacid dehydrogenase [*Lactiplantibacillus plantarum*]	0	0.997	WP_142262829.1
gene1742	1807979	1808285	-	GIY-YIG nuclease family protein [*Lactiplantibacillus plantarum*]	1E-66	1	WP_003645628.1
gene1743	1808268	1809027	-	tRNA1(Val) (adenine(37)-N6)-methyltransferase [*Lactiplantibacillus plantarum*]	0	1	WP_003645629.1

gene1744（*plsC*）	1809138	1809774	+	1-acylglycerol-3-phosphate O-acyltransferase [*Lactiplantibacillus plantarum* ST-III]	2E-153	1	ADN98865.1
gene1745	1809830	1810061	-	hypothetical protein LPST_C1650 [*Lactiplantibacillus plantarum* ST-III]	3E-48	1	ADN98866.1
gene1746	1810164	1810437	-	hypothetical protein Lp16_1605 [*Lactiplantibacillus plantarum* 16]	4E-56	1	AGO08290.1
gene1747（*lexA*）	1810555	1811188	+	LexA repressor [*Lactiplantibacillus plantarum*]	1E-152	0.9952	MCG0732895.1
gene1748	1811811	1812441	+	hypothetical protein [*Lactiplantibacillus plantarum*]	2E-142	0.9904	WP_015825658.1
gene1749	1812490	1813660	-	hydroxymethylglutaryl-CoA synthase [*Lactiplantibacillus plantarum*]	0	1	WP_015380455.1
gene1750	1813695	1814088	-	hypothetical protein [*Lactiplantibacillus plantarum*]	3E-92	1	KZT97394.1
gene1751	1814251	1814644	+	hypothetical protein [*Lactiplantibacillus plantarum*]	5E-88	0.9923	WP_015380457.1
gene1752	1815089	1816031	+	glycosyltransferase family 2 protein [*Lactiplantibacillus plantarum*]	0	0.9968	MDN6215184.1
gene1753	1816730	1817303	-	helix-turn-helix domain containing protein [*Lactiplantibacillus plantarum*]	1E-135	0.9947	WP_031275254.1
gene1754（*tagT_U_V*）	1817454	1818543	-	LCP family protein [*Lactiplantibacillus plantarum*]	0	1	MBS0953805.1
gene1755	1818581	1819013	-	putative transposase for insertion sequence element IS6501 [*Lactiplantibacillus plantarum*]	2E-102	1	OUT01805.1
gene1756	1818985	1819357	-	Transposase [*Lactiplantibacillus plantarum* ZJ316]	6E-85	1	AGE37926.1
gene1757	1819474	1820266	-	ABC transporter ATP-binding protein [*Lactiplantibacillus plantarum*]	0	0.9962	WP_260187879.1
gene1758	1820284	1822027	-	ABC transporter permease subunit [*Lactiplantibacillus plantarum*]	0	1	WP_016527118.1
gene1759（*rodA*）	1822505	1823717	-	FtsW/RodA/SpoVE family cell cycle protein [*Lactiplantibacillus plantarum*]	0	1	WP_003645640.1
gene1761	1824503	1824878	-	phage holin [*Lactiplantibacillus plantarum*]	8E-78	0.9839	WP_285298920.1
gene1762	1824890	1825154	-	hypothetical protein [*Lactiplantibacillus plantarum*]	2E-53	1	MCG0627622.1
gene1763	1825153	1826179	-	GH25 family lysozyme [*Lactiplantibacillus plantarum*]	0	0.9528	WP_172639632.1
gene1764	1826190	1826436	-	hypothetical protein [Lactiplantibacillus paraplantarum]	2E-44	0.9136	WP_056988443.1
gene1765	1826432	1827797	-	collagen-like protein [*Lactiplantibacillus plantarum*]	0	0.7362	WP_301667370.1
gene1766	1827780	1827942	-	hypothetical protein [*Lactiplantibacillus plantarum*]	8E-27	0.9811	WP_165836136.1
gene1767	1827945	1828188	-	hypothetical protein [*Lactiplantibacillus plantarum*]	3E-47	0.9875	WP_163627199.1
gene1768（*sadA*）	1828180	1830976	-	phage tail protein [*Lactiplantibacillus plantarum*]	0	0.7575	WP_262339558.1
gene1769	1830992	1833407	-	phage tail spike protein [*Lactiplantibacillus plantarum*]	0	0.9478	WP_163627195.1

Table S17. The terpene region elements of *Lactiplantibacillus plantarum* JS21.

Gene	Start	Stop	Strand	Description	E-Value	Percent Identity	Accession
gene2784	2845125	2845353	-	PLDc N-terminal domain-containing protein [*Lactiplantibacillus plantarum*]	2E-46	0.9867	WP_074161759.1
gene2785	2845413	2846097	-	metallophosphoesterase [*Lactiplantibacillus plantarum*]	2E-167	1	WP_225914106.1
gene2786(*cpdA*)	2846093	2846261	-	hypothetical protein [*Lactiplantibacillus plantarum*]	2E-31	1	WP_225914107.1
gene2787	2846702	2847254	+	Acetyltransferase [*Lactiplantibacillus plantarum* subsp. plantarum P-8]	6E-133	0.9945	AGL65399.2
gene2788	2847525	2848524	-	IS30 family transposase [*Lactiplantibacillus plantarum*]	0	0.997	WP_247965010.1
gene2789(*lrgA*)	2848781	2849201	-	CidA/LrgA family protein [*Lactiplantibacillus plantarum*]	7E-90	0.9928	WP_063852339.1
gene2790(*lrgB*)	2849220	2849718	-	LrgB family protein [*Lactiplantibacillus plantarum*]	3E-107	0.9939	MCG5037381.1
gene2791(*lrgB*)	2849748	2849949	-	LrgB family protein [*Lactiplantibacillus plantarum*]	5E-37	1	WP_134795272.1
gene2792	2850123	2850999	-	DegV family protein [*Lactiplantibacillus plantarum* ZJ316]	0	1	AGE40626.1
gene2793	2851487	2851655	-	hypothetical protein [*Lactiplantibacillus plantarum*]	2E-31	1	WP_021357416.1

gene2794	2851822	2852521	+	zinc metallopeptidase [*Lactiplantibacillus plantarum*]	3E-165	0.9957	WP_057717055.1
gene2795	2853126	2854623	+	phytoene desaturase family protein [*Lactiplantibacillus plantarum*]	0	1	WP_011102097.1
gene2796	2854603	2855485	+	phytoene/squalene synthase family protein [*Lactiplantibacillus plantarum*]	0	1	WP_027822316.1
gene2797	2855846	2856788	+	alpha/beta hydrolase [*Lactiplantibacillus plantarum*]	0	0.9968	WP_168177697.1
gene2798	2856854	2857967	-	glycerate kinase [*Lactiplantibacillus plantarum*]	0	1	WP_054519129.1
gene2799	2858102	2859437	+	NAD(P)/FAD-dependent oxidoreductase [*Lactiplantibacillus plantarum*]	0	1	WP_027822314.1
gene2800	2859631	2859961	+	bacteriocin immunity protein [*Lactiplantibacillus plantarum*]	1E-72	1	WP_021357419.1
gene2801	2860183	2861482	-	adenylosuccinate lyase [*Lactiplantibacillus plantarum*]	0	1	WP_003645480.1
gene2802	2861798	2863088	+	adenylosuccinate synthase [*Lactiplantibacillus plantarum*]	0	1	WP_015381012.1
gene2803	2863130	2864108	+	GMP reductase [*Lactiplantibacillus plantarum* ZJ316]	0	1	AGE40638.1
gene2804	2864200	2865142	-	ABC transporter ATP-binding protein [*Lactiplantibacillus plantarum*]	0	0.9968	AYG28017.1

Table S18. The cyclic-lactone-autoinducer region elements of *Lactiplantibacillus plantarum* JS21.

Gene	Start	Stop	Strand	Description	E-Value	Percent Identity	Accession
gene3001	3072566	3073772	-	FAD-dependent oxidoreductase [*Lactiplantibacillus plantarum*]	0	0.9975	WP_301699601.1
gene3002	3074238	3074610	+	TIGR02328 family protein [*Lactiplantibacillus plantarum*]	6E-87	0.9919	WP_172637956.1
gene3003	3074805	3075354	-	folate family ECF transporter S component [*Lactiplantibacillus plantarum*]	6E-124	0.9945	WP_196241767.1
gene3004	3075483	3076482	+	IS30 family transposase [*Lactiplantibacillus plantarum*]	0	0.997	WP_247965010.1
gene3005	3076664	3076799	-	hypothetical protein [*Lactiplantibacillus plantarum*]	2E-20	1	WP_003642924.1
gene3006	3076815	3077061	-	GlsB/YeaQ/YmgE family stress response membrane protein [*Lactiplantibacillus plantarum*]	2E-44	1	WP_070085348.1
gene3007 （*katE*）	3077406	3078861	+	catalase [*Lactiplantibacillus plantarum* subsp. plantarum ATCC 14917 = JCM 1149 = CGMCC 1.2437]	0	0.9979	EFK28610.1
gene3008 （*spxA*）	3079018	3079456	-	Spx/MgsR family RNA polymerase-binding regulatory protein [*Lactiplantibacillus plantarum*]	2E-101	0.9931	WP_103420511.1
gene3009 （*agrA*）	3079811	3080555	-	LytTR family DNA-binding domain-containing protein [*Lactiplantibacillus plantarum*]	0	0.9919	WP_168786167.1
gene3010	3080547	3081810	-	GHKL domain-containing protein [*Lactiplantibacillus plantarum*]	0	1	WP_054519202.1
gene3011	3081819	3081948	-	cyclic lactone autoinducer peptide [Lactiplantibacillus]	1E-21	1	WP_003643585.1
gene3012	3081928	3082525	-	accessory gene regulator AgrB [*Lactiplantibacillus plantarum*]	1E-134	0.9949	WP_241467921.1
gene3013 （*clpL*）	3082892	3085007	-	ATP-dependent Clp protease ATP-binding subunit [*Lactiplantibacillus plantarum*]	0	0.9972	WP_057137373.1
gene3014	3085397	3085631	-	hypothetical protein [*Lactiplantibacillus plantarum*]	6E-50	0.987	WP_169484439.1
gene3015 （*lctO*）	3086259	3087360	-	lactate oxidase [*Lactiplantibacillus plantarum*]	0	1	WP_102115675.1
gene3016	3087386	3089135	-	thiamine pyrophosphate-binding protein [*Lactiplantibacillus plantarum*]	0	0.9966	WP_108910153.1
gene3017	3089222	3089648	-	Rrf2 family transcriptional regulator [*Lactiplantibacillus plantarum*]	5E-99	0.9929	WP_123808678.1
gene3018 （*poxL*）	3089820	3091632	-	pyruvate oxidase [*Lactiplantibacillus plantarum*]	0	1	WP_003646223.1

gene3019（*srlB*）	3092002	3092380	-	glucitol/sorbitol-specific PTS system IIA component [*Lactiplantibacillus plantarum*]	1E-84	1	BEI65651.1

Table S19. Putative probiotic genes are found in the genome of *Lactiplantibacillus plantarum* JS21.

Gene ID	Gene Name	Strand	Start	End	KO ID	KO Description
Stress resistance genes						
gene2094	*atpC*	-	2128509	2128081	K02114	F-type H+-transporting ATPase subunit epsilon
gene2095	*atpD*	-	2129924	2128521	K02112	F-type H+/Na+-transporting ATPase subunit beta [EC:7.1.2.2 7.2.2.1]
gene2096	*atpG*	-	2130893	2129949	K02115	F-type H+-transporting ATPase subunit gamma
gene2097	*atpA*	-	2132443	2130923	K02111	F-type H+/Na+-transporting ATPase subunit alpha [EC:7.1.2.2 7.2.2.1]
gene2098	*atpH*	-	2133011	2132466	K02113	F-type H+-transporting ATPase subunit delta
gene2099	*atpF*	-	2133516	2133001	K02109	F-type H+-transporting ATPase subunit b
gene2100	*atpE*	-	2133782	2133570	K02110	F-type H+-transporting ATPase subunit c
gene2101	*atpB*	-	2134533	2133820	K02108	F-type H+-transporting ATPase subunit a
gene1587	*uup*	-	1641766	1639868	K15738	ABC transport system ATP-binding/permease protein
gene0566	-	-	591900	589918	K06158	ATP-binding cassette, subfamily F, member 3
gene0354	-	+	372316	373500	-	cation: proton antiporter
gene0651	*nhaK*	+	695509	697638	K24163	monovalent cation/hydrogen antiporter
gene0728	-	-	775397	773541	K03455	monovalent cation: H+ antiporter-2, CPA2 family
gene2241	*nhaK*	+	2270261	2272264	K24163	monovalent cation/hydrogen antiporter
gene2293	*nhaK*	-	2321931	2320339	K24163	monovalent cation/hydrogen antiporter
gene2426	-	-	2468631	2467465	-	cation: proton antiporter
gene2691	*nhaK*	-	2747753	2746152	K24163	monovalent cation/hydrogen antiporter
gene2862	*nhaC*	-	2927387	2925963	K03315	Na+:H+ antiporter, NhaC family
gene0123	-	-	130332	127594	-	MULTISPECIES: cation-transporting P-type ATPase
gene0945	*ctpE*	+	1011030	1013369	K12952	cation-transporting P-type ATPase E [EC:7.2.2.-]
gene1627	-	-	1689159	1686883	K01535	H+-transporting ATPase [EC:7.1.2.1]
gene2632	*copA*	-	2677369	2675444	K17686	P-type Cu+ transporter [EC:7.2.2.8]
gene2855	-	+	2917466	2919310	-	MULTISPECIES: heavy metal translocating P-type ATPase
pWS05b0022	-	+	22708	25482	-	HAD-IC family P-type ATPase
DNA and protein protection and repair						
gene0618	*clpP*	-	650100	649510	K01358	ATP-dependent Clp protease, protease subunit [EC:3.4.21.92]
gene0840	*clpC*	+	897938	900442	K03696	ATP-dependent Clp protease ATP-binding subunit ClpC
gene1000	*clpE*	-	1066996	1064930	K03697	ATP-dependent Clp protease ATP-binding subunit ClpE
gene1125	*clpP*	+	1178947	1179666	K01358	ATP-dependent Clp protease, protease subunit [EC:3.4.21.92]
gene1613	*clpB*	-	1671411	1668808	K03695	ATP-dependent Clp protease ATP-binding subunit ClpB
gene1780	*clpP*	-	1845110	1844346	K01358	ATP-dependent Clp protease, protease subunit [EC:3.4.21.92]
gene1865	*clpX*	-	1903396	1902131	K03544	ATP-dependent Clp protease ATP-binding subunit ClpX
gene3013	*clpL*	-	3085007	3082893	K04086	ATP-dependent Clp protease ATP-binding subunit ClpL
Exopolysaccharide biosynthesis responsible genes						
gene0228	*cysE*	-	244105	243563	K00640	serine O-acetyltransferase [EC:2.3.1.30]
gene0406	*glmU*	+	421185	422567	K04042	bifunctional UDP-N-acetylglucosamine pyrophosphorylase / glucosamine-1-phosphate N-acetyltransferase [EC:2.7.7.23 2.3.1.157]

gene0450	-	+	476535	478139	K03328	polysaccharide transporter, PST family
gene0555	*galE*	+	579356	580351	K01784	UDP-glucose 4-epimerase [EC:5.1.3.2]
gene0572	*wecA*	+	598014	599111	K02851	UDP-GlcNAc:undecaprenyl-phosphate/decaprenyl-phosphate GlcNAc-1-phosphate transferase [EC:2.7.8.33 2.7.8.35]
gene0594	*galU*	+	620532	621452	K00963	UTP--glucose-1-phosphate uridylyltransferase [EC:2.7.7.9]
gene0677	*licD*	-	725651	724842	K07271	lipopolysaccharide cholinephosphotransferase [EC:2.7.8.-]
gene0963	*wecB*	+	1029283	1030401	K01791	UDP-N-acetylglucosamine 2-epimerase (non-hydrolysing) [EC:5.1.3.14]
gene1049	-	+	1122380	1124050	-	polysaccharide biosynthesis protein
gene1287	*pgaC*	-	1342525	1341212	K11936	poly-beta-1,6-N-acetyl-D-glucosamine synthase [EC:2.4.1.-]
gene1440	-	+	1491247	1491852	K21713	lytic chitin monoxygenase [EC:1.14.99.53]
gene1843	-	-	1885579	1884575	-	polysaccharide biosynthesis C-terminal domain-containing protein
gene1852	*galE*	-	1892059	1891118	K01784	UDP-glucose 4-epimerase [EC:5.1.3.2]
gene1855	*epsA*	-	1893757	1893560	K19420	protein tyrosine kinase modulator
gene1856	*epsA*	-	1894320	1893760	K19420	protein tyrosine kinase modulator
gene1887	*pgaC*	-	1927958	1926606	K11936	poly-beta-1,6-N-acetyl-D-glucosamine synthase [EC:2.4.1.-]
gene2111	*manA*	-	2146653	2145688	K01809	mannose-6-phosphate isomerase [EC:5.3.1.8]
gene2327	*pgaC*	+	2362543	2363871	K11936	poly-beta-1,6-N-acetyl-D-glucosamine synthase [EC:2.4.1.-]
gene2969	*galE*	-	3037290	3036286	K01784	UDP-glucose 4-epimerase [EC:5.1.3.2]
pWS03b0015	*epsA*	+	12532	13299	K19420	protein tyrosine kinase modulator
pWS03b0055	*epsA*	+	54095	54199	K19420	protein tyrosine kinase modulator
gene1854	*epsB*	-	1893542	1892835	K00903	protein-tyrosine kinase [EC:2.7.10.3]
pWS03b0016	*epsB*	+	13310	14038	K00903	protein-tyrosine kinase [EC:2.7.10.3]
Lipoteichoic acid (LTA) synthesis responsible genes						
gene1706	*dltD*	-	1767288	1766011	K03740	D-alanine transfer protein
gene1708	*dltB*	-	1768765	1767551	K03739	membrane protein involved in D-alanine export
Adhesion ability						
gene1524	-	-	1574724	1573018	-	fibronectin-binding domain-containing protein
gene0427	*srtA*	+	445842	446546	K07284	sortase A [EC:3.4.22.70]
Anti-pathogenic effect						
gene0609	*luxS*	+	638874	639350	K07173	S-ribosylhomocysteine lyase [EC:4.4.1.21]
Biofilm formation						
gene0018	*glgC*	+	23093	24232	K00975	glucose-1-phosphate adenylyltransferase [EC:2.7.7.27]
gene0019	*glgC*	+	24229	25401	K00975	glucose-1-phosphate adenylyltransferase [EC:2.7.7.27]
gene0020	*glgA*	+	25394	26833	K00703	starch synthase [EC:2.4.1.21]
gene0021	*glgP*	+	26853	29249	K00688	glycogen phosphorylase [EC:2.4.1.1]
gene0228	*cysE*	-	244105	243563	K00640	serine O-acetyltransferase [EC:2.3.1.30]
gene0609	*luxS*	+	638874	639350	K07173	S-ribosylhomocysteine lyase [EC:4.4.1.21]
gene0620	*rpoN*	+	651074	652411	K03092	RNA polymerase sigma-54 factor
gene0711	*crr*	+	758720	759211	K02777	sugar PTS system EIIA component [EC:2.7.1.-]
gene0963	*wecB*	+	1029283	1030401	K01791	UDP-N-acetylglucosamine 2-epimerase (non-hydrolysing) [EC:5.1.3.14]
gene1287	*pgaC*	-	1342525	1341212	K11936	poly-beta-1,6-N-acetyl-D-glucosamine synthase [EC:2.4.1.-]
gene1398	*trpE*	+	1453129	1454565	K01657	anthranilate synthase component I [EC:4.1.3.27]
gene1399	*trpG*	+	1454516	1455124	K01658	anthranilate synthase component II [EC:4.1.3.27]

gene1887	*pgaC*	-	1927958	1926606	K11936	poly-beta-1,6-N-acetyl-D-glucosamine synthase [EC:2.4.1.-]
gene2327	*pgaC*	+	2362543	2363871	K11936	poly-beta-1,6-N-acetyl-D-glucosamine synthase [EC:2.4.1.-]
gene2575	*crr*	-	2625805	2625359	K02777	sugar PTS system EIIA component [EC:2.7.1.-]
gene2786	*cpdA*	-	2846261	2846094	K03651	3',5'-cyclic-AMP phosphodiesterase [EC:3.1.4.53]
pWS05 0001	*glgC*	+	3	392	K00975	glucose-1-phosphate adenylyltransferase [EC:2.7.7.27]
pWS05 0002	*glgA*	+	389	1831	K00703	starch synthase [EC:2.4.1.21]
pWS05 0003	*glgP*	+	1838	4252	K00688	glycogen phosphorylase [EC:2.4.1.1]
pWS05 0035	*glgC*	+	38120	39268	K00975	glucose-1-phosphate adenylyltransferase [EC:2.7.7.27]
pWS05 0036	*glgC*	+	39255	40424	K00975	glucose-1-phosphate adenylyltransferase [EC:2.7.7.27]
pWS05 0037	*glgA*	+	40421	41863	K00703	starch synthase [EC:2.4.1.21]
pWS05 0038	*glgP*	+	41870	44284	K00688	glycogen phosphorylase [EC:2.4.1.1]

Table S20. The hydrophobicity, auto-aggregation, and co-aggregation of *Lactiplantibacillus plantarum* JS21

hydrophobicity	auto-aggregation	Co-aggregation			
		E. coli (ATCC 25922)	*E. coli* (K88)	*S. aureus* (CMCC 26003)	*L. monocytogenes* (CICC 21635)
60.04±0.96%	42.63±1.06%	31.69±0.51%	30.97±0.83%	44.92±0.30%	35.35±0.70%

Table S21. Tolerance of JS21 to simulated gastrointestinal fluids

Simulated gastric fluid(pH=2.0)	Simulated Intestinal Fluid(pH=6.8)		Simulated gastric fluid(pH3.0)	Simulated Intestinal Fluid(pH=6.8)	
3h Survival rate (%)	3h Survival rate (%)	6h Survival rate (%)	3h Survival rate (%)	3h Survival rate (%)	6h Survival rate (%)
18.91%	173.72%	244.06%	143.75%	182.19%	205.48%

Table S22. JS21 Inhibition zone results of CFS against pathogens bacteria

Test Strain	Strain Code	Zone of Inhibition (±SD) (mm)			
		CFS	NC	Ampicillin	Kanamycin
Escherichia coli	ATCC 25922	16.35±0.04	0	11.17±0.07	19.85±0.04
Escherichia coli	K88	13.53±0.15	0	12.82±0.05	16.46±0.10
Staphylococcus aureus	CMCC 26003	16.78±0.11	0	31.72±0.14	17.43±0.03
Listeria monocytogenes	CICC 21635	14.80±0.08	0	10.8±0.04	18.51±0.04
